# The IGS-ETS in *Bacillus *(Insecta Phasmida): molecular characterization and the relevance of sex in ribosomal DNA evolution

**DOI:** 10.1186/1471-2148-8-278

**Published:** 2008-10-09

**Authors:** Andrea Ricci, Valerio Scali, Marco Passamonti

**Affiliations:** 1 Dipartimento di Biologia Evoluzionistica Sperimentale, University of BolognaVia Selmi 3, 40126 Bologna, Italy

## Abstract

**Background:**

DNA encoding for ribosomal RNA (rDNA) is arranged in tandemly-repeated subunits, each containing ribosomal genes and non-coding spacers. Because tandemly-repeated, rDNA evolves under a balanced influence of selection and "concerted evolution", which homogenizes rDNA variants over the genome (through genomic turnover mechanisms) and the population (through sexuality).

**Results:**

In this paper we analyzed the IGS-ETS of the automictic parthenogen *Bacillus atticus *and the bisexual *B. grandii*, two closely related stick-insect species. Both species share the same IGS-ETS structure and sequence, including a peculiar head-to-tail array of putative transcription enhancers, here named *Bag530*. Sequence variability of both IGS-ETS and *Bag530 *evidenced a neat geographic and subspecific clustering in *B. grandii*, while *B. atticus *shows a little but evident geographic structure. This was an unexpected result, since the parthenogen *B. atticus *should lack sequence fixation through sexuality. In *B. atticus *a new variant might spread in a given geographic area through colonization by an all-female clone, but we cannot discard the hypothesis that *B. atticus *was actually a bisexual taxon in that area at the time the new variant appeared. Moreover, a gene conversion event between two *Bag530 *variants of *B. grandii benazzii *and *B. grandii maretimi *suggested that rRNA might evolve according to the so-called "library hypothesis" model, through differential amplification of rDNA variants in different taxa.

**Conclusion:**

On the whole, *Bacillus *rDNA evolution appears to be under a complex array of interacting mechanisms: homogenization may be achieved through genomic turnover that stabilizes DNA-binding protein interactions but, simultaneously, new sequence variants can be adopted, either by direct appearance of newly mutated repeats, or by competition among repeats, so that both DNA-binding proteins and repeat variants drive each other's evolution. All this, coupled with chromosome reshuffling due to sexuality (when present), might drive a quick fixation of new rDNA variants in the populations.

## Background

Ribosomal RNA genes (rDNA) encode the RNA scaffold of the ribosome and thereby perform what likely is the most basic of all housekeeping functions. Within the rDNA cluster, each repeat contains one copy of the 18S, 5.8S and 28S rRNA genes, which are separated by several spacers: two internal transcribed spacers, ITS1 (between 18S and 5.8S) and ITS2 (between 5.8S and 28S), an external transcribed spacer (ETS, between promoter and 18S), and an untranscribed intergenic spacer (IGS), separating each adjacent rDNA unit. The rDNA unit is transcribed by the RNA polymerase I (RNA-PolI) as a single 45S precursor molecule, which is subsequently processed by an ordered cleavage of the spacers. The rDNA units are organized in large tandem arrays (rDNA loci), generally located within one or a few chromosome pairs, where they constitute the nucleolar organizer regions (NOR). During active synthesis, rDNA loci are associated to the nucleolus (for a review see [1]).

While the core domain of the rDNA coding region is well conserved, the IGS is rapidly evolving, so that it has been used as a marker for identification of closely related species and for quantification of gene flow between populations ([2] and references therein). In eukaryotes, tandemly repeated multigene families, like rDNA, undergo a peculiar pattern of evolution known as "concerted evolution" [3]: as a consequence of this, the repeats show a higher sequence homogeneity within a species (or population) than between species (or populations). This pattern is achieved through two independent mechanisms known as homogenization (*i.e. *the spreading of a variation to the whole repeat array) and fixation (*i.e. *the fixation of the variant within a taxonomic unit). Homogenization is achieved through several genomic turnover mechanisms, such as unequal crossing-over and gene conversion, while fixation is a by-product of the chromosome reshuffling due to sexual reproduction [3].

Despite the presence of molecular mechanisms that homogenize the ribosomal arrays, variations in rDNA have been commonly observed both within and among populations of several species. This variation is both quantitative and qualitative, the former consisting in a redundancy of rDNA copies and in the number of loci per genome, the latter being typically due to the occurrence of regulatory sub-repeats within the rDNA cluster [4]. Actually, the IGS contains the RNA-PolI promoter and other important regulatory elements, such as terminators, spacer promoters and enhancers. As a consequence, the IGS region may show a marked variation in length, due to the redundancy of those functional elements (repeats) [5,6]. Moreover, a higher number of duplicated regulatory elements has been related to an increased rate of rDNA transcription, which seems to play an adaptive role ([2] and references therein).

While the overall structural organization of the RNA-PolI machinery is comparable in all eukaryotes, the regulatory repeats are quite variable within each species, and hardly comparable between species, so that the region associated with the rDNA transcription promoter is generally species-specific (*i.e*., the RNA-PolI complexes of one species fail to transcribe the rRNA genes of another) ([7] and references therein). Consequently, IGS and RNA-PolI transcription factors seem to co-evolve in a species-specific way; therefore, this appears as a good candidate system for analyses on evolutionary co-adaptation of gene complexes [8]. As a matter of fact, selection must be taken into account to understand rDNA dynamics, so that, on the whole, the evolution of rDNA clusters is likely under a balanced influence of selection and "concerted evolution". In more detail, selection likely acts differently, the coding regions being more likely under purifying selection, whereas the IGS may experience more relaxed selective pressure [4].

Then, the repeated organization of rDNA would allow the spreading of new variations trough the mechanisms of genomic turnover, as well as the sexual reproduction could allow to fix them in the reproductive units (*i.e*. "concerted evolution"). In this scenario, any mutation in the controlling region, which would produce a better interaction to an essential transcription factor, should be selected for and fixed by unequal crossing over and gene conversion events during gametogenesis, as well as in somatic cells trough mitotic recombination [9]. As a consequence of those mechanisms, the rDNA clusters, as well as their dedicated transcription machinery, would experience rapid evolutionary changes, which could lead to a rapid evolution of post-mating isolation mechanisms. All that considered, the rDNA cluster represents a good candidate for studies on speciation. With this in mind, we started a characterization of the rDNA cluster of the Mediterranean genus *Bacillus*, which comprises two bisexual species (*Bacillus grandii *and *Bacillus rossius*), three unisexual parthenogenetic species (*Bacillus atticus, Bacillus whitei *and *Bacillus lynceorum*), and two hybridogenetic strains (*Bacillus rossius-grandii benazzii *and *Bacillus rossius-grandii grandii*). On the whole, *Bacillus *taxa constitute a clear case of reticulate evolution (*i.e. *the exploitation of a wide array of reproductive modes leading to a net of phyletic links) and provide a good experimental system to analyze the multifaceted relationships among sexual and metasexual taxa [10,11].

This paper deals with the description of the IGS-ETS in *B. atticus *and *B. grandii*, two closely related species, as evidenced by mitochondrial and satellite DNA analyses ([10,11] and references therein). *B. grandii *– a strict bisexual taxon – shows a relic disjunct distribution in Sicily, and is divided into three subspecies, *B. grandii grandii, B. grandii benazzi *and *B. grandii maretimi*. Unlike *B. grandii, B. atticus *– at present an all-female obligate parthenogen – shows a much wider distribution in the western Mediterranean basin, and forms a complex of three different karyological (2n = 32; 2n = 34; 3n = 48–51) and allozymic races, without morphological diversification. Based on this, *B. atticus *has been divided into three subspecies, namely *B. atticus atticus, B. atticus carius *and *B. atticus cyprius *[10,11].

The characterization of *Bacillus *IGS-ETS, the first known for a phasmid insect, allowed us to infer about the variability and evolution of this peculiar tandemly-repeated system, and to compare it to the additional IGS-ETSs known to date in arthropods. Moreover, patterns of evolution of rDNA repeated sequences in *Bacillus *could be easily comparable to what has been observed for *Bacillus *pericentromeric satellite DNA. Actually, the *Bacillus *system represents an intriguing model to investigate how "concerted evolution" works on repeated sequences, because the existence of either apomictic and automictic parthenogens provides a unique opportunity to study the effects of meiosis and sex in multigene family evolution [10,11]. In more detail, the automictic parthenogen *B. atticus*, although maintaining meiosis and crossing over, lacks chromosome reshuffling due to sexual reproduction: for this reason, and according to the "concerted evolution" rationale, new variants can easily spread within a single *B. atticus *female and her progeny (*i.e. *a clone of parthenogens), but cannot spread in the whole taxonomic unit. On the other hand, the bisexual *B. grandii*, being the closest relative of *B. atticus*, represents the perfect counterpart, since in this species the repeated sequences would follow the "concerted evolution".

## Methods

### IGS characterization

Specimens of *Bacillus atticus*, *B. grandii grandii, B. g. benazzi *and *B. g. maretimi *were collected at several Mediterranean locations. Taxa, localities and sample acronyms are reported in Table 1.

**Table 1:** *rDNA *characterization in *Bacillus*

Taxon	locality	acromyms	GenBank A.N.	
			*Bag530*-*PstI *clones	^3'^IGS-ETS
			
*Bacillus atticus atticus (2n = 34, ♀ ♀)*	Palaeochora, Greece	AAT/Pal	4 [EU855066–EU855070]	[EU855050]
	Castel di Tusa, Italy	AAT/Tus	9 [EU855071–EU855079]	[EU855049]
	Golan, Israel	AAT/Isr	12 [EU855054–EU855065]	[EU855048]*
*Bacillus atticus cyprius (2n = 32, ♀ ♀)*	Episkopi, Cyprus	ACY/Epk3	4 [EU855090–EU855093]	
		ACY/Epk4	6 [EU855094–EU855099]	[EU855046]
*Bacillus atticus carius (3n = 51, ♀ ♀)*	Neraida, Greece	ACA/Ner	10 [EU855080–EU855089]	[EU855047]
*Bacillus grandii grandii (2n = 34, ♀ ♂)*	Cava Grande del Cassibile, Italy	GG/Cag28	1 [EU855111]	-
		GG/Cag137	2 [EU855112–EU855113]	[EU855051]
		GG/Cag139	4 [EU855114–EU855117]	-
*Bacillus grandii benazzii (2n = 34, ♀ ♂)*	Torre Bennista, Italy	GB/Tbe4	6 [EU855105–EU855110]	[EU855052]
*Bacillus grandii maretimi (2n = 34, ♀ ♂)*	Marettimo Is., Italy	GM/Mar1	5 [EU855100–EU855104]	[EU855053]

Species, localities, sample acronyms and GenBank A.N. of the analyzed specimens.

♀ ♀ = all female parthenogen; ♀ ♂ = bisexual;

* = Clone obtained by sequencing the portion of *Pst*I_5500 _from the last *Bag550 *repeat to the 5'-end of the 18S gene.

Field-collected specimens were stored at -80°C. Total genomic DNA was isolated from somatic tissues with a standard phenol-chloroform protocol.

Since we didn't have any specific PCR primer to amplify the whole rDNA cluster, we decided to proceed with a wide restriction enzyme screening using the following enzymes: *Taq*, *Alu*, *Sma*, *Ava*, *HpaI*, *HpaII*, *ClaI*, *SstI*, *StuI*, *CfoI*, *HhaI*, *MspI*, *DraI*, *HincII*, *XbaI *and *PstI*. Such exploratory restriction analysis was performed on specimens of *B. atticus *from Castel di Tusa (Italy), Neraida (Greece) and Israel. *XbaI *produced a ladder-like pattern (the shorter fragment at about 350 bp, here referred to as *XbaI*_350_), while *PstI *produced two main fragments, a small one of 530 bp and a high molecular weight one of 5.5 kb (here referred to as, respectively, *PstI*_530 _and *PstI*_5500_). The ladder-like pattern of the *XbaI *fragments evidenced that this sequence is tandemly repeated. Total genomic DNA from *B. atticus *and *B. grandii *specimens (see Table 1) were then digested using *PstI *in order to obtain *PstI*_530 _and *PstI*_5500 _fragments.

Fragments were separated by agarose gel electrophoresis and transferred into a nylon membrane by Southern blotting. Two probes were used for hybridization: the first was the *XbaI*_350 _fragment, the latter was a PCR-obtained portion of the 18S gene (5'-end), amplified with the following universal primers: 18S-s22(F) 5'-TAATGATCCTTCCGCAGGTTCA-3'; 18S-A1984(R) 5'-TCCCTGGTTGATCCTGCCAGTA-3', using Herculase II fusion DNA polymerase according to manufacturer's instructions. The probes were digoxigenine-labeled and hybridization was performed at 65°C accordingly to DIG DNA Labeling and Detection Starter Kit I (Roche); filters were washed with low stringency buffer (0.5 × SSC, 0.1% SDS), corresponding to ~80% sequence similarity between probe and target.

The fragments *XbaI*_350_, *PstI*_530 _and *PstI*_5500 _from *B. atticus *from Israel (AAT/Isr) were cloned into a pGem-7Zf(+) plasmid (Promega) vector using the *E. coli *DH5α competent cells (Invitrogen). 64 *PstI*_530 _clones were also obtained by restriction from *B. grandii *and *B. atticus *specimens (see Tab. 1). Recombinant clones were screened by PCR using M13 universal primers. Some of the obtained recombinant colonies were purified using *SNAP Miniprep *kit (Invitrogen) according to the manufacturer's instructions.

Clones were sequenced using M13 universal primers by Macrogen Inc. The portion upstream the 18S gene of the *PstI*_5500 _fragment was sequenced using "primer walking" method in *B. atticus *from Israel (AAT/Isr) (Table 1), and this allowed us to determine the IGS structure in *B. atticus*.

The *PstI*_530 _fragment was also used as a probe for in situ hybridization according to [12] on *B. atticus *chromosomes.

Given the observed structure of the IGS-ETS (see Results), and to perform a large comparison of IGS-ETSs among *Bacillus *species with a reasonable sequencing effort, we decided not to sequence the whole *PstI*_5500 _fragment for all *B. grandii *and *B. atticus *specimens, but to focus our attention on the 3' portion of the IGS and the ETS, from the last *PstI*_530 _repeat and the 5' of the 18S gene (a region here referred to as ^3'^IGS-ETS). To obtain these fragments, the PCR was performed with *Herculase *(Stratagene) following the manufacturer's instructions. A primer (Bag(F)-5'-ACAGGCAAATGGGAGTTG-3') was designed on the *PstI*_530_consensus sequences. This primer was then coupled with the 18S universal primer by [13], modified as follows 18i(R)-5'-TTTCTCAGGCTCCCTCTCCGGAATCGAACCCT-3'. The PCR products were amplified and completely sequenced using the "primer walking" method as described above. Analyzed specimens and GenBank Accession numbers are reported in Table 1.

### Sequence analyses

All sequences were aligned using the CLUSTAL algorithm of the Sequence Navigator Software (Applera) and corrected by eye. Phylogenetic analyses on *PstI*_530 _and ^3'^IGS-ETS were performed using Neighbor-Joining (NJ), Maximum Parsimony (MP) and Maximum Likelihood (ML) using PAUP* v.4.0b [14]. Likelihood scores for each DNA substitution model were estimated using MODELTEST and used for subsequent analyses [15]. The best-scored model was JC+Γ for *PstI*_530 _and F81+Γ for IGS-ETS sequences. Support for each node was obtained using bootstrap (500 replicates) [16]. Jukes-Cantor nucleotide distance values and nucleotide composition were calculated with MEGA 4.0 [17]. Nucleotide diversity (Pi) distribution across *PstI*_530 _clones and ^3'^IGS-ETS were calculated with a 30 and 60 bp sliding window and step size of 15 and 10 bp respectively, using DnaSP program v.4.1 [18]. The conserved and variable regions were considered significant when Pi exceed 2 × SD (standard deviation) the average Pi, as described in [19].

Secondary structures were analyzed with the MFOLD server program [20]. To search for the presence of any internal repeat, the ^3'^IGS-ETS sequences were analyzed using the server program REPFIND [21]. The Neural Network Promoter Prediction tool [22] was used to identify potential promoter sequences.

## Results

Endonuclease restriction analysis of *Bacillus atticus *showed restriction fragments for *XbaI *and *PstI*: *XbaI *produced a ladder-like pattern, with the shorter band at about 350 bp, (*XbaI*_350_), which indicates the presence of a tandemly repeated DNA. *PstI *produced two main fragments, one of about 530 bp (*PstI*_530_), the other at about 5.5 kb (*PstI*_5500_).

**Figure 1:**
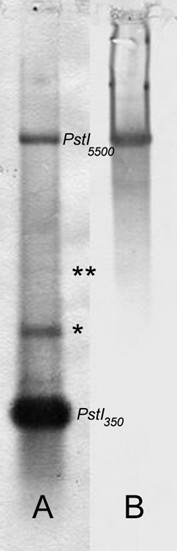
**Southern blot**. Southern blot of the *PstI *restriction pattern in *B. atticus*, using *XbaI*_350 _(A) and the PCR-obtained 5'-end of the 18S gene (B) as hybridizing probes. Southern Blot analysis revealed that: *i) XbaI*_350_and *PstI*_530 _cross-hybridize, with *XbaI*_350_being part of the *PstI*_530 _fragment (also tested by subsequent sequencing); *ii) PstI*_530 _is a tandemly repeated sequence (* = *PstI*_530 _dimer; ** = *PstI*_530 _trimer), as also supported by *XbaI*_350_restriction pattern (not shown); *iii*) 18S PCR probe and *XbaI*_350 _hybridize with *PstI*_5500_, so they are associated; *iv*) the *PstI*_5500 _band is the rDNA cluster, since it hybridizes to the 18S probe (also checked by subsequent sequencing).

Southern blot analysis (Fig. 1) and sequencing unequivocally revealed that *XbaI*_350 _is part of the *PstI*_530 _sequence, henceforth named *Bag530*.

**Figure 2:**
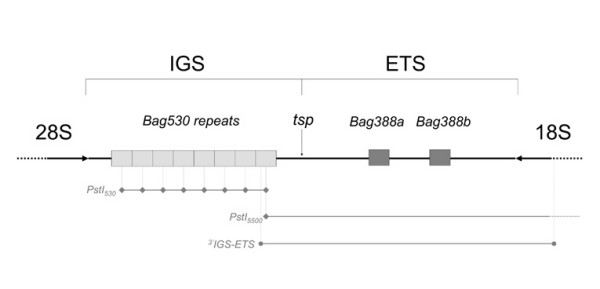
**The IGS-ETS of *Bacillus *stick insects**. Schematic drawing of the IGS-ETS structure of *Bacillus *stick insects. The IGS region is characterized by a large array of tandemly repeated head-to-tail sequences, here named *Bag530*, that are proposed to be RNA-PolI enhancers. Downstream the last *Bag530 *sequence, the transcription start point (*tsp*) mark the IGS-ETS boundary. Redundant *tsp *sequences are also found in each *Bag530 *enhancer. The ETS show a twofold 388 bp direct repeats (*Bag388a *and *Bag388b*), with an unknown regulatory function. Sequenced fragments are reported in gray: fragments obtained by *PstI *restriction analysis (*PstI*_530 _and *PstI*_5500_) are marked with gray rhombs (◆); ^3'^IGS-ETS fragment, obtained by PCR, is marked with gray circles (●).

Southern blot analysis also evidenced that the *PstI*_5500 _fragment is part of the ribosomal DNA, containing both sequences of the 18S gene and *Bag530 *(*i.e. *both *XbaI*_350_/*PstI*_530 _fragments)(Figure 1). Direct sequencing evidenced that the *PstI*_5500 _fragment in *B. atticus *from Israel includes part of the last *Bag530 *monomer at the 5' end, while at the 3'ends downstream the 18S gene, therefore including the full ETS sequence (Figure 2). Moreover, *in situ *hybridization using *PstI*_530 _as a probe evidenced that it localizes in the NOR of *B. atticus *(data not shown). *Bag530 *and ^3'^IGS-ETS fragments were then sequenced in *B. atticus *and *B. grandii *subspecies, as reported in Table 1.

### Structural organization of the IGS-ETS in *Bacillus*

In *B. atticus *and *B. grandii*, the average nucleotide content of ^3'^IGS-ETS sequences is 52.9% A+T (range 52.5%–53.8%), while the 374 bp belonging to the 5'-end of the structural 18S gene show 53.1% A+T content. On the whole the average length of the ^3'^IGS-ETS is 2583 bp, including 347 bp of the 18S gene (see additional file 1 material for ^3'^IGS-ETS alignment and annotation).

Figure 2 summarizes the structure of the IGS-ETS region in *Bacillus*, as determined by sequencing the *PstI*_5500 _and ^3'^IGS-ETS fragments. The exact position of 5' end of 18S was established by aligning *Bacillus *sequence to the 18S sequence of *Blattella germanica *[GenBank:AF005243]. The IGS-ETS region showed the typical basic structure with both repetitive arrays and non-repetitive sequences. Repetitive regions are of two main types: a large cluster of head-to-tail repeats, corresponding to the *Bag530 *array, and a twofold 388 bp direct repeat (named *Bag388a *and *Bag388b *respectively) spaced by a 191 bp unique sequence. From the ^3'^IGS-ETS alignment (see additional file 2 data) it is evident that the last monomer of the *Bag530 *is differently truncated, namely at the position 280 in *B. atticus *and at the 260 in *B. grandii*. The first unique sequence (590 bp long) characterizes the 5' of the IGS-ETS, downstream the *Bag530 *cluster. The *Bag388a *repeat is located in the position 871/1258, while the *Bag388b *is between 1446/1835. The two *Bag388a *and *Bag388b *repeats are separated by a sequence of 191 bp and followed by a unique sequence (428 bp long) upstream the 5' end of 18S.

Although the boundary between the IGS and ETS has not being actually determined, we found a motif similar to the transcription start point (*tsp*) sequence of other arthropods, downstream the *Bag530 *cluster. The observed motif (5'-TATATTAGAGGGA-3') well matches to the promoter consensus sequence (5'-TATA>TANGRRRR-3') of several arthropods (see additional file 3 data; [23]), and it has been also confirmed by using the Neural Network Promoter Prediction tool, which predicted the same sequence (5'-TTTTGGGTATATTAGAGGGA-3') with a score of 0.93. Assuming that we did find the real gene promoter, ETS is therefore 1736 bp long in *B. atticus *and 1727 bp long in *B. grandii*. This length is quite different from what typically found in other arthropods, where the ETS regions are usually 500–1000 bp long [2], with few exceptions such as *Daphnia pulex *(1280 bp) [23].

### The *Bag530 *repeat

As evidenced by restriction analysis and sequencing, the IGS region of *Bacillus *is characterized by an arrays of head-to-tail tandemly-repeated *Bag530 *repeats. In this study we sequenced 64 *Bag530 *repeats obtained from restriction analysis from *B. grandii *and *B. atticus *(see Table 1 for details). *Bag530 *consensus sequence of 531 bp was obtained by aligning the 64 sequences; we observed a total of 42 indels, 11 of which representing a large deletion in all *B. grandii grandii *clones (see additional file 3 data). Average sequence length is 516 bp, with variants ranging from 505 bp (GG/Cag137-c2) to 520 bp (GB/Tbe4-c4 and c5).

Closer sequence examination revealed that *Bag530 *repeats could be further divided into three shorter subunits of 119 bp (A), 276 bp (B) and 120 (A'), respectively: A and A' subunits showed 73.2% of sequence similarity between them. Due to the tandemly-arranged structure of *Bag530*, and to the fact that repeat boundaries are usually defined arbitrarily by the restriction site, we could argue that the functional sequence of *Bag530 *subrepeats should actually be *B-A'-A*, because the last *Bag530 *monomer of the array appears to be truncated just downstream the A subrepeat (see above). Moreover, a putative promoter sequence (5'-TATATAGGGGGT-3') occurs in each of the *Bag530 *repeats, suggesting that these sequences are actually spacer promoters. This hypothesis is also supported by the presence of a palindromic motif of 28 bp in each repeat (5'-CCCGGCGATCGAGGCCTCGATCGCCGGG-3'), 88 bp upstream the putative spacer promoter sequence. In fact, the local fold symmetry created by the palindrome is thought to provide the binding site for DNA-binding proteins that are often dimeric, like the UBF factor involved in the machinery of the RNA-PolI [24].

### Variability of ^3'^IGS-ETS and *Bag530 *sequences

In the automictic parthenogen *B. atticus *the overall mean distances value of ^3'^IGS-ETS is 0.010 ± 0.002. The distance values among the distinct allozymic and karyological races (see Background) show the same order of magnitude when comparing *B. atticus cyprius *vs.*B. atticus carius *(0.016 ± 0.003) and *B. atticus cyprius *vs. *B. atticus atticus *(0.012 ± 0.002), while between *B. atticus atticus *and *B. atticus carius *the value is lower (0.007 ± 0.001). On the other hand, the bisexual *B. grandii *shows a nearly 6-fold higher overall ^3'^IGS-ETS distance value (0.058 ± 0.005) when compared to the unisexual *B. atticus*. Comparing the different *B. grandii *subspecies among themselves, the distance values range from 0.039 ± 0.004 (*B. grandii maretimi *vs. *B. grandii benazzi*) to 0.083 ± 0.005 (*B. grandii benazzi *vs. *B. grandii grandii*).

**Figure 3:**
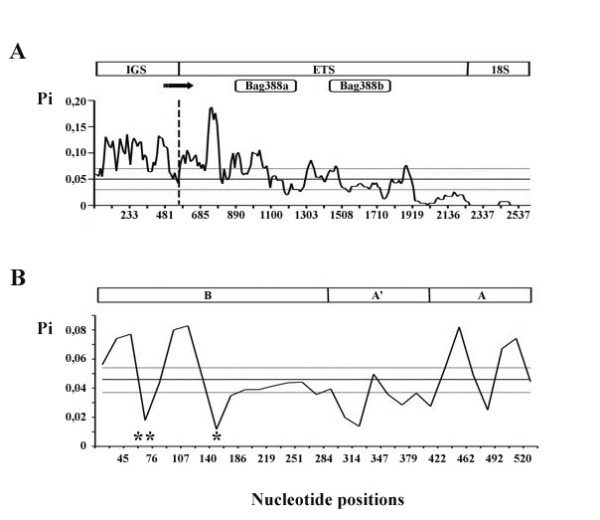
**Nucleotide variability distribution**. A) Distribution of nucleotide variability along the ^3'^IGS-ETS sequences in *Bacillus *by sliding window analysis (window size, 60 bp; step size, 10). The vertical dashed line indicates the IGS/ETS boundary. Above, the structure of ^3'^IGS-ETS sequence and schematic drawing of functional elements are reported: arrow and solid blocks indicate putative gene promoter and *Bag388*, respectively. B) Distribution of nucleotide variability along the *Bag530 *monomers using sliding window analysis (window size, 30 bp; step size, 15). One (*) and two (**) stars mark the position of the putative promoter and the palindromic sequences, respectively, which occur in each of the *Bag530 *repeats. Above, the *Bag530 *monomer structure has been represented. In both A and B sections, nucleotide positions (midpoint of sliding window) and nucleotide diversity are reported on the X and Y axes, respectively; average variability of Pi is shown by a solid line, dashed lines represent average variability ± 2 SD (standard deviation).

Variability is not evenly distributed along the ^3'^IGS-ETS sequence: the sliding window analysis evidenced that nucleotide diversity progressively drops when approaching the *18S *gene, which is likely due to selective sweep (Figure 3A). Moreover, where the putative *tsp *gene promoter was located, the only observed variation involves a single substitution (G > A) in *B. grandii benazzii *sample (5'-TATATTAGAAGG-3'). The observed high level of sequence conservation in this region gives further evidence about its structural and functional role for transcription.

The pattern of overall variability of *Bag530 *evidenced in *B. atticus *unisexuals is considerably lower to that of the sexual *B. grandii *(0.018 ± 0.003 and 0.045 ± 0.006, respectively). The values of variability between *B. atticus *subspecies are: *B. atticus cyprius *vs.*B. atticus carius *(0.022 ± 0.005), *B. atticus cyprius *vs. *B. atticus atticus *(0.018 ± 0.003), *B. atticus atticus *and *B. atticus carius *(0.019 ± 0.003). Comparing the *B. grandii *subspecies, the distance values are: *B. grandii maretimi *vs. *B. grandii benazzi *(0.021 ± 0.005), *B. grandii benazzi *vs. *B. grandii grandii *(0.072 ± 0.011), and *B. grandii grandii *vs.*B. grandii maretimi *(0.078 ± 0.012).

An homogeneous range of variability was found within the populations of *B. atticus atticus *(Paleochora, 0.013 ± 0.004; Castel di Tusa, 0.012 ± 0.003 and Israel, 0.015 ± 0.003) and *B. atticus carius *(Neraida, 0.009 ± 0.003) and *B. atticus cyprius *(Episkopi, 0.009 ± 0.002). Also within *B. grandii grandii *and *B. grandii benazzi*,*Bag530 *has similar levels of variability (0.010 ± 0.003 and 0.007 ± 0.002, respectively), while *B. grandii maretimi *showed a distance value equal to 0.015 ± 0.003, so that this subspecies seems at the first glance to be more variable than the others. However, it should be noted that such higher value is mainly due to a single *Bag530 *clone, only GM/Mar1-c3 (see Discussion): excluding it from the analysis, the level of variability falls to the value observed for *B. grandii grandii *and *B. grandii benazzi *(0.010 ± 0.003).

It is interesting to note that sequence variability is not uniformly distributed in *Bag530*: two minima fall within the B subunit, one in the region including the palindromic motif of 28 bp (see above), and the other in the region including the RNA-PolI promoter (Figure. 3B). Actually, the promoter-like sequence shows no variation among all clones, as it has been observed in other organisms. It has been supposed that this sequences have the potential to form strong secondary structures suggesting that the region may be under functional constraints.

### Phylogenetic analysis

Neighbor Joining, Maximum Parsimony and Maximum Likelihood trees based either on *Bag530 *clones or ^3'^IGS-ETS sequences showed the same basic topology, with clones/sequences of *B. atticus *and *B. grandii *falling into two major distinct clades, well supported by bootstrap values. Here, for brevity, we report only Maximum Likelihood trees.

**Figure 4:**
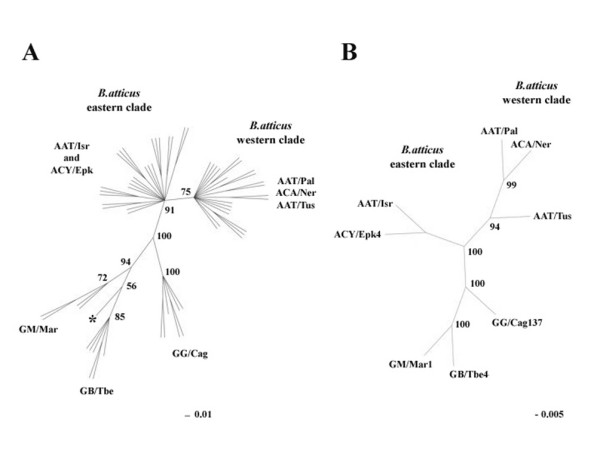
**Phylogenetic analysis**. A) Maximum Likelihood unrooted cladogram obtained from *Bag530 *monomers. The recombinant clone GM/Mar1-c3 has been marked by a star (*). B) Maximum Likelihood unrooted cladogram obtained from ^3'^IGS-ETS sequences. Numbers at nodes are bootstrap values. The bars below the trees indicate mutational steps.

Trees based on *Bag530 *sequences (Figure 4A) showed two major distinct clades: one is given by *B. grandii *sequences, the other including *B. atticus*. Within *B. atticus*, clones grouped into two well-supported clusters: the first includes *Bag530 *clones of specimens collected in the western part of the species range (*B. atticus carius *and *B. atticus atticus *from Italy and Greece), while the second includes clones from specimens living in the Eastern Mediterranean (*B. atticus cyprius *from Cyprus and *B. atticus atticus *from Israel). Within each of the two *B. atticus *clusters, clones are completely intermingled, without any hint of geographical or population trend.

The *B. grandii *clones fall into three distinct clades, one for each subspecies, with *B. grandii benazzi *and *B. grandii maretimi *being more related. A single clone obtained from *B. grandii maretimi *(GM/Mar1C3) shows a peculiar clustering, being more similar to *B. grandii benazzii *variants (Fig. 4A). By comparing clones from *B. grandii benazzii *and *B. grandii maretimi*, 12 diagnostic positions were recognized: GM/Mar1C3 shows the first nine diagnostic sites typical of *B. grandii benazzii*, while the last 3 are of *B. grandii maretimi*; therefore GM/Mar1C3 may be the result of a gene conversion.

The tree based on ^3'^IGS-ETS sequences (Fig. 4B) shows the same basic topology as that based on *Bag530 *clones.

## Discussion

### Structure of the IGS in *Bacillus*

The molecular characterization of the IGS-ETS region showed that *Bacillus atticus *and *B. grandii *share the same basic structure and sequence (Fig. 2), with only minor variations; this result strongly supports the close phylogenetic relationship between the two species, as already indicated by previous mitochondrial and allozyme analyses ([10] and references therein).

As already mentioned, IGS-ETS sequences are fast evolving, and sequence homologies can be quickly lost; however, the observed structure of *Bacillus *IGS-ETS maintains several characteristics in common with other Metazoans. In detail, several organisms, such as *Xenopus*, *Daphnia*, mouse, rat, hamster, etc. ([5,6], and references therein) show tandem repeated sequence arrays downstream the 28S gene: for those the role of RNA-PolI transcription enhancers has been proposed [5]. *Bacillus *IGS shows repetitive elements of two main types: a large cluster corresponding to the *Bag530 *sequences and the twofold *Bag388 *direct repeat. Downstream the *Bag530 *cluster, a conserved putative promoter sequence has been found, which could represent the RNA-PolI promoter, and the same promoter is also present in each *Bag530 *repeat. Therefore we are quite confident in interpreting the *Bag530 *repeats as RNA-PolI enhancers.

Although no real sequence similarity could be detected, IGS enhancers must share the same mechanism of action, which likely is in their secondary structure; for instance, it has been observed that *Xenopus *enhancers work very well in mammals and in plants. Because of this common action, coupled with no obvious sequence similarity, enhancers could function as the binding sites of a common transcriptional factor, which most likely is the UBF (for a review see [5,6], and references therein).

The presence of the *Bag383a *and *Bag383b *repeats in the ETS appears to be unique of *Bacillus *so, although they might have some sort of regulatory significance, nothing more precise can be stated at present.

Finally, the *Bacillus *rRNA at the junction between ETS and 18S show the ability to form stem-loop structures, as observed in *Drosophila melanogaster *[25] and *Apis mellifera *[25], thus suggesting that this feature is likely important for an exact recognition of the rRNA splicing site. The stem-loop structure is formed by the conserved rRNA helices H9, H17 and H9' flanking the ETS region [26] (see additional file 4 material).

### Pattern of evolution of the IGS-ETS repeats and the "concerted evolution"

The pattern of variability of *Bag530 *is quite different in *B. atticus *and *B. grandii*. In *B. atticus *some degree of geographic structure is observed, while *B. grandii *shows a more strong geographic and subspecies-specific clustering. This is also confirmed by the whole ^3'^IGS-ETS sequence analysis.

In more detail, a few *Bag530 *and ^3'^IGS-ETS variants are present in *B. atticus*, although, in theory, the absence of the homogenizing force of sexuality would permit the maintenance of many divergent variants in unisexuals. Moreover, *Bag530 *and ^3'^IGS-ETS sequences evidenced the presence of two clusters in *B. atticus*, respectively including eastern (Cyprus and Israel) and western populations (Italy and Greece), regardless of subspecies, allozymic race or karyotype they belong to (see Table 1).

As a matter of fact, the presence of a slight geographic structuring of *B. atticus Bag530 *might be considered as an unexpected result, since, according to the "concerted evolution" rationale, the automictic *B. atticus *lacks chromosome reshuffling coupled with Mendelian reproduction, and this should prevent "spreading" and "fixation" of variants in the taxonomic unit. In *Bacillus Bag320 *satellite DNA the lack of either geographic or racial clustering was explained this way [27].

How can we explain this result for *Bag530*? First of all, it is relevant to remember that different kinds of selective pressures on tandemly repeated arrays produce different background in which molecular drive is fixing newly occurring variants. Although "concerted evolution" applies to IGS-ETS too, a substantial proportion of newly arisen variants may be eliminated by selection, because this region contains promoters and enhancers interacting with RNA-PolI machinery [28]. This would likely turn in the low variability observed, even in the absence of the homogenizing contribution of sexuality. Compared to rDNA, the satellite *Bag320 *family of *Bacillus *likely undergoes a more relaxed selection, so that unisexuals can maintain more variability in satellite DNA.

All that considered, we may speculate that the slight geographic structuring of *B. atticus *IGS-ETS might be an outcome of geographically-based co-evolution of the IGS-ETS RNA-PolI machinery. With this in mind, we still need to explain how newly arisen IGS-ETS variants might spread in a large geographical area, in absence of chromosome reshuffling due to sexual reproduction. We can suggest that populations carrying the new variant might have spread from a founding clone of *B. atticus *females, in which the new variant has been fixed by selection and genomic turnover mechanisms allowed by automictic parthenogenesis. However, another possibility must be mentioned, *i.e. *that by the time the new variant appeared, *B. atticus *was still a bisexual taxon. This hypothesis is not unrealistic, since it has been proved that in Sicily, at the time that the tri-hybrid *B. lynceorum *arose from *B. rossius *females (about 1 Myr ago), *B. atticus *males had to be present to form the hybrid [11]. At present, however, *B. atticus *males have never been observed in nature, although the species has been intensively collected.

In *B. grandii*, the pattern of variability of *Bag530 *and ^3'^IGS-ETS might be explained by bisexuality acting as a driving force on repeated sequence homogenization/fixation in the same genomic pool (molecular drive), coupled with the co-evolution of the rDNA arrays within each gene pool. The result is a clear racial clustering of the *Bag530 *repeats, which is fully in line with what has been observed for the *Bag320 *Satellite DNA family [27]. Moreover, the invention of a recombinant clone between two *Bag530 *variants of *B. grandii benazzii *and *B. grandii maretimi *must be an outcome of the coexistence of both variants in the same genome, either now in the genome of *B. grandii maretimi*, or in a common ancestor of the subspecies, which are nowadays no longer sympatric. A very similar recombination event has been observed in the *Bag320 *satellite DNA of *B. rossius *[29].

Studies on satellite DNAs evidenced the occurrence of the so-called "library hypothesis" based on the "expansion-contraction" model: related taxa could share low-copy repetitive DNA sequences that can be specifically amplified in each evolutionary unit, thus becoming highly represented in one taxon while remaining at a low copy number in the others [30,31]. In agreement with the "library hypothesis", Mestrovic et al. [32] have recently proposed that rapid changes in satellite DNA profiles (within/between single family of satellite DNAs) could be achieved through replacements of functionally equivalent repeats of DNA, without affecting the DNA-proteins binding. The existence of specific profiles as a consequence of copy number changes in a set of satellite DNAs shared by related genomes was found in species of the insects *Palorus *and *Pimelia *[33,34],*Bacillus *[29] and in root-knot nematodes [32].

Therefore, the *Bag530 *GM/Mar1C3 clone might be in line with the "library hypothesis" of repeated DNA, since the observed recombination event should have occurred when both variants were present in the same genome; so we can speculate that rDNA enhancers, and likely the IGS-ETS itself, might experience the same "expansion-contraction" mechanism described for other repeated DNAs.

## Conclusion

All that considered, rDNA might be constrained by a complex array of evolutionary mechanisms: repeats homogenization achieved through genomic turnover mechanism may stabilize interactions with DNA-binding protein thus eliminating or limiting the effects of deleterious mutations (stable complex) but, simultaneously, the whole array could acquire new sequence variants, better fitting the above mentioned interactions (flexible system). The co-evolution can in turn be driven in a dual way, either by direct acquisition of newly mutated repeats, or by competition among repeats that better fit to the above mentioned DNA-proteins interactions. In this way, both DNA-binding proteins and repeat variants would drive each other's evolution leading to a new protein/DNA pair to replace the old one [35]. All this, coupled with sexuality might drive quick fixation of new rDNA variants in populations.

## Authors' contributions

AR carried out the molecular genetic studies, sequence alignment, statistical analysis and drafted the manuscript. VS participated in the design of the study and helped drafting the manuscript. MP conceived of the study, participated in its design and coordination and helped to draft the manuscript. All authors have read and approved the final manuscript.

## Supplementary Material

Additional file 1 **Sequence alignment of the ^3'^IGS-ETS sequences in *Bacillus***. Sequence alignment and annotation of the ^3'^IGS-ETS sequences in *Bacillus*. Species acronyms as in Table 1. Putative functional sequences are marked in bold. The stem and loop structure at the boundary between ETS and 18S gene is reported above the sequence at the appropriate position of the alignment. Click here for file

Additional file 2 **Sequence alignment of the *Bag530 *sequences in *Bacillus***. Sequence alignment of the *Bag530 *sequences in *Bacillus*. Species acronyms as in Table 1. The "C" followed by a number indicates the number of clone sequenced. Click here for file

Additional file 3 **Putative promoter sequences comparisons**. Putative promoter sequence of *B. atticus *and *B. grandii *(Ba-g). Promoter sequences of *Triops cancriformis *(Tca), *Daphnia pulex *(Dpu) and *Artemia franciscana *(Afr) were also reported for comparison. The first nucleotide of the promoter sequence is indicated by (>) and seven bp of the upstream sequence are reported; (g) and (s) indicate gene and spacer promoters respectively. Consensus sequence of regions surrounding rDNA *tsp *of several arthropods is reported, as described by Crease (1993). Click here for file

Additional file 4 **Secondary structure of the ETS-18S junction**. A) Secondary structure of the ETS-18S junction. Calculated free energy is reported below the structure. B) Conserved rRNA helices (H9, H17 and H9') flanking 3'-end ETS are within dashed vertical line Click here for file
