# Supplementary material for: The IGS-ETS in Bacillus (Insecta Phasmida): molecular characterization and the relevance of sex in ribosomal DNA evolution
Source: BMC Evol Biol. 2008 Oct 9;8:278. doi: 10.1186/1471-2148-8-278 (PMC2590618; doi:10.1186/1471-2148-8-278)
Supplement: Additional file 1 — Sequence alignment of the 3'IGS-ETS sequences in Bacillus. Sequence alignment and annotation of the 3'IGS-ETS sequences in Bacillus. Species acronyms as in Table 1. Putative functional sequences are marked in bold. The stem and loop structure at the boundary between ETS and 18S gene is reported above the sequence at the appropriate position of the alignment. [file 1471-2148-8-278-S1.pdf]

Additional file 1 – Sequence alignment and annotation of the [3'] IGS-ETS sequences in *Bacillus*

```
                   10         20         30         40         50         60         70         80         90        100
ACY/Epk4  ATGAAAAATC TCCCGGCGGC CGAGGCCTCC ACACCCCGGG ACGCTCCTGG CGCACGGACT TTGTGTCGGC ACCTCGCTGC AGCCATGTGG GAGTAGAAAA
ACA/Ner   .......-.. .........T .......... .......... .......... .......... .......... .......... .......... ..........
AAT/Isr   .......... .......... .......... -......... .......... .......... .......... .......... .......... ..........
AAT/Tus   .......... .........T .......... -......... .......... .......... .......... .......... .......... ..........
AAT/Pal   .......... .........T .......... -......... .......... .......... .......... .......... .......... ......G...
GG/Cag137 .......... ........AT .......... .......... .A.G...... .......... .......T.. .GTG...... ......T... C.........
GB/Tbe4   G..T...... ........AT .......... .........C .A.G...... .......... .......T.. ..T....... ...A...... ..........
GM/Mar1   G..T...... ........AT .......... .........C GA.G...... .......... .......T.. ..T....... ...A...... ..........
          ----------------------------------------------------------------------------------------------------------
          ----------------------------------------------------------------------------------------------------------
```

Bag530

```
                  110        120        130        140        150        160        170        180        190        200
ACY/Epk4  TGTTAAATTC CGCAGGCCTG ATAATTCTCC CGGCGATGGA GGCCTCCGCA CCCCGGGAAA TCCCGG-CGC CCGGACTTTG TGCGAGCCCC TCGCAGCATC
ACA/Ner   .......... .......... .A........ .......... .......... .......... .....T-... .......... ..T....... .AT..T..G.
AAT/Isr   .......... .......... .......... .......... .......... .......... ......-... .......... .......... ..........
AAT/Tus   .......... .......... .A........ .......... .......... .G........ ......-... .......... ..T....... ........G.
AAT/Pal   .......... .......... .A........ .......... .......... .......... .....T-... .......... ..T....... .AT..T..G.
GG/Cag137 .......AAA .AG....TA. .A..A..... .......... .......... .A........ AA..T.G... ..C....... ..T.C...T. ........CA
GB/Tbe4   ......TAAA .AA....TAA .A........ .......... A......... .......... AA..T.G... .......... ..T.C..... ..C..A..A.
GM/Mar1   ......TAAA .AG....TAA .A........ .......... ......G... .......... AA..T.G... .......... ..T.C..... ........G.
          ----------------------------------------------------------------------------------------------------------
          ----------------------------------------------------------------------------------------------------------
```

Bag530

```
                  210        220        230        240        250        260        270        280        290        300
ACY/Epk4  CGAGCGAAAC AGCCGAAATG AAAGGAGCGG GGAGCCAAAA AGTTTCTCCC GGCGATCGAG GCCTCGA-TC GCCGGGGAAA TCTCCGGCGC CTGGGCTCGC
ACA/Ner   .......... .......... .......... .......... .A........ ........G. .......-.. .......... .......... .C........
AAT/Isr   .......... .......... .......... .......... .......... .......... .......-.. .......... .......... ..........
AAT/Tus   .......... .......... .......... .......... .A........ ........G. .......-.. .......... .......... .C........
AAT/Pal   T......... .......... .......... .......... .A........ ........G. .......-.. .......... .......... .C........
GG/Cag137 .A........ .......... .......... ..CC.TTG.. .AA....... ........G. ...A...C.G C..------- ---------- ----------
GB/Tbe4   .A........ .......... ...C...... ..CC.T.... .AA....... ........T. ...G..GCAG C..------- ---------- ----------
GM/Mar1   .A........ ....A..... ...C...... ..CC.T.G.. .AA....... ........T. ...G..GCAG C..------- ---------- ----------
          ---------------------------------------------------------------------------------->
          ------------------------------------------------------------>

                  310        320        330        340        350        360        370        380        390        400
ACY/Epk4  CGTGCCCTCG AAATCCTGCG AAGCTCTGAC TTTGCCTGTG ATGCAGTTCC CATTATGTCA AGCGTGACAA T--GTTTTAA AAAA-ATTCT GTTCGTGGTT
ACA/Ner   .......... .......... .......... .......... .......... .......... ...C...... .--....... ....-..... ..........
AAT/Isr   .......... .......... .......... .......... .......... .......... ...C...... .--....... ....-..... ..........
AAT/Tus   .......... .......... .......... .......... .......... .......... ...C...... .--....... ....-..... ..........
AAT/Pal   .......... .......... .......... .......... .......... .......... ...C...... .--....... ....-..... ..........
GG/Cag137 ---------- -------... .....G.A.. .....G.... ..T.G.G... ......A... ...C..G... .AG....... ....T..A.. ..........
GB/Tbe4   ---------- -------... .....G.... ....G...G. .AT.C.G... ......A..C ...C...... .AA.....T. .G..AT..A. .G........
GM/Mar1   ---------- -------... .....G.... .......... ..T.G.G... ......A... ...C...... .AG.....T. .G..AT..A. ..........
```

```
                  410        420        430        440        450        460        470        480        490        500
ACY/Epk4   GTTTGTAAAC AACTGCGATT TTGGAATTCA ACCCATTGTC TATGATCATG TACCCAGAGT TACCCCCTTC TGTTTCAGTT ATAATCTGAT TTCCTGGGAA
ACA/Ner    .......... .......... .....T.... .......... .......... .........G .......... .......... .......... ..........
AAT/Isr    .......... .......... .......... .......... .......... .......... .......... .......... .......... ..........
AAT/Tus    .......... .......... .....T.... .......... .......... .........G .......... .......... .......... ..........
AAT/Pal    .......... .......... .....T.... .......... .......... .........G .......... .......... .......... ..........
GG/Cag137  .......... .......... .......... .....A.AG. .........T .....GA..G ..A..G.CCA ...A...... .A........ ..........
GB/Tbe4    .G........ C...GC.... .........C ........C. G.A......T .....G..AG ..A...T.C. A......... .A.......A ..........
GM/Mar1    .......... .......... .......... ........C. G.C......T .....G...G ..A...T.C. A......... .A........ ..........

                  510        520        530        540        550        560        570        580        590        600
ACY/Epk4   TCAGTGTCTA CCTATCGATA GGCCCATTTT GGGTATATTA GAGGGACATG CAGTTGGTGA AGAATTGGAA TTGTTTGCAA TATGAATATA ATGTGTATAC
ACA/Ner    .......... .......... .......... .......... .......... .......... .......... .......... .........T ..........
AAT/Isr    .......... .......... .......... .......... .......... .......... .......... .......... .......... ..........
AAT/Tus    .......... .......... .......... .......... .......... .......... .......... .......... .........T ..........
AAT/Pal    .......... .......... .......... .......... .......... .......... .......... .......... .........T ..........
GG/Cag137  A......T.. .A........ .......... .......... .......... .......G.- .A........ ...A...... ......ATAT .........A
GB/Tbe4    A.....GT.. A.C....... A......... .......... ..A....... ...G.T.... .A..A..... ...G....C. ......AT.. ........TA
GM/Mar1    A......T.. ..CT...... .......... .......... .......... .......... .A........ .......... ......ATA. .........A
                                    <--------------->
                                          tsp

                  610        620        630        640        650        660        670        680        690        700
ACY/Epk4   TGAATAAAAA GGGAGTCCCT CCGAGAATGC TCGCCCTTAA ATATTGCAAA AATTTCACAG AAAAAATAAT GTGGCAAGTA GAGCACCTCG CGGCAAAGCG
ACA/Ner    .......... .......... .......... .......... .......... .......... .......... .......... .......... ..........
AAT/Isr    .......... .......... .......... .......... .......... .......... .......... .......... .......... .......CT.
AAT/Tus    .......... .......... .......... .......... .......... .......... .......... .......... .......... ..........
AAT/Pal    .......... .......... .......... .......... .......... .......... .......... .......... .......... ..........
GG/Cag137  .........- .......... ......T.T. .......... .......... .T.....T.. .....T..T. .......... .........A ..........
GB/Tbe4    ..G....... -..G...... T...A.T.T. ..CG...... ...A...... .TAA.A.T.. ....-G.... .....C.... ..A..A.... .......A..
GM/Mar1    .........- ...G...... ......T.T. .......... .......... .TA..A.T.. .....G..T. .......... .......... ..........

                  710        720        730        740        750        760        770        780        790        800
ACY/Epk4   GACATGCAAC CTCTGAGAAT GGTTTTGAAT GCTCCTACTT CAGTTTCCCT TAGAGGCGGA CAATACAAAA TGTGATTTAT ATAGTGATTG GCTCTTGCAT
ACA/Ner    .......... .......... ......A... .......... ..T....... .......... ....G..... .....A.... .......... ..........
AAT/Isr    .......... .......... .......... .......... .......... .......... .......... .......... .......... ..........
AAT/Tus    .......... .......... .......... .......... .......... .......... ....G..... .....A.... .......... ..........
AAT/Pal    .......... .......... ......A... .......... .......... .......... ....G..... .....A.... .......... ..........
GG/Cag137  .......... ..T....... ...A...... .......... TC..A...TA A.AG.TG.AT G.CCGA.... .....A.... ........A. ..........
GB/Tbe4    .......... -..C..A... ..AA.-.G.. ........C. A.......AA A.A....AA. ..C.TG.... ....GA.... ........A. ...TC..G..
GM/Mar1    .......... .......... ...A...... .......... A.......AA A.AG.CA.AC G.C.GA.... .....A.... ........A. ..........
```

```
                     810        820        830        840        850        860        870        880        890        900
ACY/Epk4      ATGGTGACTT AACCGTCTGG GAAAGTAGGC TGCAATTTAT TTAGAAGGTT GTATGTTGTC TGTTACACTG TGGAGGTGCT CAAGAGACCC GTGGAAGTCC
ACA/Ner       .......... .......... .......... .......... .........A .......... .......... C......... .T........ ..........
AAT/Isr       .......... .......... .......... .......... .......... .......... .......... .......... .......... ..........
AAT/Tus       .......... .......... .......... .......... .......... .......... .......... .......... .T........ ..........
AAT/Pal       .......... .......... .......... .......... .......... .......... .......... .......... .T........ ..........
GG/Cag137     .......... ..GT.C.... .......... .......... ..G....... .......... .......... .A........ .GT....... A..T......
GB/Tbe4       .........- .......... .......T.. ..-....... .CT-..T... .......... .......... .AC....... .GT....T.A .....T....
GM/Mar1       .........- .......... .......T.. ..-....... .CT-..T... .......... .......... .AC....... .GT....T.A .....T....
                                                                                      <-------------------------------

                     910        920        930        940        950        960        970        980        990       1000
ACY/Epk4      AAGCAGAAAT ATTCTAAAAG AGGGTGAGGT CTTCATTCTG CCTTGGCTTG TGGTCTCAGT TTTATGGAAG ACCAGCCCTC CCTCTCGTGG CAATGGGTGC
ACA/Ner       .......... ..-A...... .......... ......-... .......... .......... ..-....... .......... .......... ......T...
AAT/Isr       .......... .......... .......... .......... .......... .......... .......... .......... .......... ..........
AAT/Tus       .......... .......... .......... .......... .......... .......... .......... .......... .......... ......T...
AAT/Pal       .......... .......... .......... .......... .......... .......... .......... .......... .......... ......T...
GG/Cag137     .......... .......... .......... .......... .......... .......... .......... .......... ..G....... ..........
GB/Tbe4       .......... G...T.T... ........T. ....C..... ........G. G..-.....C ...TA..... ....A...CT ..C...C... GC.....G..
GM/Mar1       .......... G...T.T... .......... .......... .T........ .........C .......... .......... ..G....... ..........
              ------------------------------------------------------------------------------------------------------------
                                                           Bag338a

                    1010       1020       1030       1040       1050       1060       1070       1080       1090       1100
ACY/Epk4      TTAGGTGAAC ATTTATGGCT GTATGATATA TTGGAAACTA AGAAAAAAAC CTTGTAGCCA TGAACTGTGC ATACTAAATA CTATCCCGTG TGGCTTGTCT
ACA/Ner       .......... .......... .......... .......... ........G- .......T.. .......... .......... ........A. ..........
AAT/Isr       .......... .......... .......... .......... .......... .......... .......... .......... .......... ..........
AAT/Tus       .......... .......... .......... .......... ........G. -......T.. .......... .......... ........A. ..........
AAT/Pal       .......... .......... .......... .......... ........G. -......T.. .......... .......... ........A. ..........
GG/Cag137     ......G... ...C...... ........-. .-...G.... ..C.....G. -.......A. .......... .C.A...G.. ........A. ....A.....
GB/Tbe4       ...A...GGA C..CC..... .G.....A.. ......-... GCC....-G. -.......A. .......... .C........ ........A. ....A.....
GM/Mar1       ......G... G..C...... .......... .......... ..C.....G. -.......A. .......... .C........ ........A. ....A.....
              ------------------------------------------------------------------------------------------------------------
                                                           Bag338a

                    1110       1120       1130       1140       1150       1160       1170       1180       1190       1200
ACY/Epk4      GTACTCAATA CACTAGTCGC GAGCTATTGC TATACTTTTG TCTGGTAATG AGCGACGATT CGATGGGATG CGAAATGGTG GGGAACGCGT GTCCTGTCGT
ACA/Ner       .......... .......... .......... .......... .......... .......... .......... .......... .......... ..........
AAT/Isr       .......... .......... .......... .......... .......... .......... .......... .......... .......... ..........
AAT/Tus       .......... .......... .......... .......... .......... .......... .......... .......... .......... ..........
AAT/Pal       .......... .......... .......... .......... .......... .......... .......... .......... .......... ..........
GG/Cag137     .......... .......... .G..C.A... ....T.G..- --...G.... .......... .......... .......... .....T...C ..........
GB/Tbe4       .......... .......... ......A... ..A.T..... .......... .......... .......... .......... .........C A.........
GM/Mar1       .......... .......... ....C.A... ..A.T..... .....G.... .......... .......... .......... .........C A.........
              ------------------------------------------------------------------------------------------------------------
                                                           Bag338a
```

```
                 1210       1220       1230       1240       1250       1260       1270       1280       1290       1300
ACY/Epk4   CTTAAACATT GATGGTTCCC CTCAGTACAT AATGAAATGG TTGGTGGATC GCAGCTCCTT GAGGAAAATG TTCCAAATGG GTTTAGTCGC GGTCTTCAGG
ACA/Ner    .......... .......... .......... .......... .......... .......... .......... .......... .......... ..........
AAT/Isr    .......... .......... .......... .......... .......... .......... .......... .......... .......... ..........
AAT/Tus    .......... .......... .......... ...A...... .......... .......... .......... .......... .......... ..........
AAT/Pal    .......... .......... .......... .......... .......... .......... .......... .......... .......... ..........
GG/Cag137  .......... .......... .......... .CG......T ...A...... .......... .......... .......... .......G.. .........T
GB/Tbe4    .......... .......... .......... .........T ...A...... .......... .......... .......... .......... .........T
GM/Mar1    .......... .......... .......... .........T ...A...... .......... .......... .......... .......... .........T
           -------------------------------------------------------------->

                 1310       1320       1330       1340       1350       1360       1370       1380       1390       1400
ACY/Epk4   CTGGGCGAAG TTTGTTCTTG AAGTGGAATT AGAAAAAAAA --ATGATGGG AAGTAGAGCA CCTTGCAGGA TAGCGGACAT GCAACCTATG ATATATGATT
ACA/Ner    .......... ....G..... .......... ........-- --........ .......... .......... .......... .......... ..........
AAT/Isr    .......... .......... .......... .......... A--....... .......... .......... .......... .......... ..........
AAT/Tus    .......... ....G..... .......... .........- ---....... .......... .......... .......... .......... ..........
AAT/Pal    .......... ....G..... .......... .........- ---....... .......... .......... .......... .......... ..........
GG/Cag137  T.....C..T ....G.T..A .......G.. T......... -T....A... ...C.A.... ...C...... ...T...... .......... .A.....T..
GB/Tbe4    T.....C... ....G..... .......G.. T........T AG....A... ...C...... ...C...... .......... .......... .G.....T..
GM/Mar1    T.....C... ....G..... .......G.. T........T AG....A... ...C...... ...C...... .......... .......... .G.....T..

                 1410       1420       1430       1440       1450       1460       1470       1480       1490       1500
ACY/Epk4   AAATGCTCCT ACATAATATC AGAAAGTTGT GTTTTTGATA CCCTGTGGTG GTGCTCAAGA GACCCGTGGA AGTCCAAGCA GAAATATTCT AAAAGAGGGT
ACA/Ner    T......... .......... .......... .......... .......... .......... .......... .......... .......... ..........
AAT/Isr    .......... .......... .......... .......... .......... .......... .......... .......... .......... ..........
AAT/Tus    T......... .......... .......... .......... .......... .......... .......... .......... .......... ..........
AAT/Pal    T......... .......... .......... .......... .......... .......... .......... .......... .......... ..........
GG/Cag137  T......... .....C.T.. .......... .......... ..T.....A. .....T.CT. ....A..... .......... ........T. ..........
GB/Tbe4    T......... .....C.T.. .......... .......... ..T.....A. .....T.GT. .......... .....TT... .......... ..........
GM/Mar1    T......... .....C.T.. .......... .......... ..T.....A. .....T.GT. .......... .....TT... ........T. ....T.....
                                                            <-----------------------------------------------------------

                 1510       1520       1530       1540       1550       1560       1570       1580       1590       1600
ACY/Epk4   GAGGTCTTCA TTCTGCCTTG GCTTGTGGTC TCAGTTTTAT GGAAGACCAG CCCTCCCTCT CGTGGCAATG GGTGCTTAGG TGAACGTTTA TGGCTGTATG
ACA/Ner    .......... ..T....... .......... .......... .......... .......... .......... .......... .......... ..........
AAT/Isr    .......... .......... .......... .......... .......... .......... .......... .......... .......... ..........
AAT/Tus    .......... .......... .......... .......... .......... .......... .......... .......... .......... ..........
AAT/Pal    .......... .......... .......... .......... .......... .......... .......... .......... .......... ..........
GG/Cag137  .....T.... ..T....... .......... ....C..... .......... .......G.. .......... ....G..... .....A..C. ..........
GB/Tbe4    .......... .......... .......... ....C..... .......... .......G.. .......... .......... ..G.....C. ..........
GM/Mar1    .......... ..T....... .......... ....C..... .......... .......G.. .......... ....G..... ..G.....C. ..........
           -------------------------------------------------------------------------------------------------------------
```

Bag338b

```
                 1610       1620       1630       1640       1650       1660       1670       1680       1690       1700
ACY/Epk4   ATATATTGGA AACTAAGAAA AAAGCTTGTA GCCATGAACT GTGCATACTA AATACTATCC CGAGTGGCTT GT-CTGTACT CAATACACTA GTCGCGAGCT
ACA/Ner    .......... .......... .......... .......... .......... .......... .......... ..-....... .......... ..........
AAT/Isr    .......... .......... .......... .......... .......... .......... .......... ..G....... .......... ..........
AAT/Tus    .......... .......... .......... .......... .......... T......... .......... ..G....... .......... ..........
AAT/Pal    .......... .......... .......... .......... .......... .......... .......... .-G....... .......... ..........
GG/Cag137  .......... ......C... .C........ ..A....... ....C..... .......... ........A. ..-....... .......... .....T...C
GB/Tbe4    .......... ......C... .......... ..A....... ....C..... .......... ........A. ..-....... .......... .........C
GM/Mar1    .......... ......C... .......... ..A....... ....C..... .......... ........A. ..-....... .......... .........C
           ---------------------------------------------------------------------------------------------------------
```

Bag338b

```
                 1710       1720       1730       1740       1750       1760       1770       1780       1790       1800
ACY/Epk4   ATTGATATAC TTTTGTCTGG TAATGAGCGA CGATTCGATG GGATGCGAAA TGGTGGGGAA CGCGTGTCCT GTCGTCTTAA CGAATGATGG TTCCT-CTCA
ACA/Ner    ....C..... .......... .......... .......... .......... .......... .......... .......... .C........ .....-....
AAT/Isr    ....C..... .......... .......... .......... .......... .......... .......... .......... .......... .....-....
AAT/Tus    ....C..... .......... .......... .......... .......... .......... .......... .......... .C........ .....G....
AAT/Pal    ....C..... .......... .......... .......... .......... .......... .......... .......... .C........ .....-....
GG/Cag137  .A..C....T .......... G......... .......... .......... .......... TT........ .......... .......... ....C-....
GB/Tbe4    .A..C..... .......... .......... .......... .......... .......... .......... .......... .......... ....C-....
GM/Mar1    .A..C..... .......... .......... .......... .......... .......... .......... .......... .......... ....C-....
           ---------------------------------------------------------------------------------------------------------
```

Bag338b

```
                 1810       1820       1830       1840       1850       1860       1870       1880       1890       1900
ACY/Epk4   GTACACAATG AAATTTTCG- ATGGATCGCA GCTCCCTGAG AGACGTGTTT CAAAGGGGTT TGTTGCGGTC TTTAGTCCGG CCAAGGTGTT GGGG--AGTA
ACA/Ner    .......... .........- .......... .......... .......... .......... A......... ........T. G.C.A.GTG. t...G-....
AAT/Isr    .......... .........- .......... .......... .......... .......... .......... ........T. G.C.A.GTG. T...--....
AAT/Tus    .......... .........T .......... .......... .......... .......... A......... ........T. G.C.A.GTG. T...GC....
AAT/Pal    .......... .........T .......... .......... .......... .......... A......... ........T. G.C.A.GTG. T...G-..-.
GG/Cag137  ....G..... ....G....- .......... .......... ....T..... .......... A..C...... ........T. G.C.A.GTG. TT..GG....
GB/Tbe4    ...TG..... ....G....- .......... .......... ....T..... ....T..... A..C...... ........T. GTC.A.GTG. T-..GG....
GM/Mar1    ...TG..... ....G....- .......... .......... ....T..... ....T..... A..C...... ........T. GTC.A.GTG. T-..GG....
           ------------------------------------->
```

```
                 1910       1920       1930       1940       1950       1960       1970       1980       1990       2000
ACY/Epk4   AAAACTTTTT GGGCCAGTGA CAATGCCCAG ACCACA-GCG AGCCCATGGG CCAACGGTCT GCTCCCGCAG TGGGCCG-TG CCC-GAACAT GGGCACCGAT
ACA/Ner    .....C.... .......... .......-.. ......-... .......... .......... .......... .......-.. ...-...... ..........
AAT/Isr    .....C.... .........- .......-.. ......A... .......... .......... .......... .......-.. ...-...... ..........
AAT/Tus    ....-C...- -........- .......-.C ..-.---... ......-... .......... .....-.... ...C.-.--. ..-G...... ..........
AAT/Pal    .....C.... -......... .......-.. ......-... .......... .......... .......... .......G.. ...C...... ..........
GG/Cag137  .....C.... .......... .......-.. ......-... .......... .......... .......... .......-.. ...-...... ..........
GB/Tbe4    .....C.... .......... .......-.. ......-... .......... .......... .......... .......-.. ...-...... ..........
GM/Mar1    .....C.... .......... .......-.. ......-... .......... .......... .......... .......-.. ...-...... ..........
```

```
                2010       2020       2030       2040       2050       2060       2070       2080       2090       2100
ACY/Epk4  CA-TACATAT GAAATCCACT CGCCATGTAC C-ATGCGAGT TTTTAAGAGC GCGATTATTC GCAAGTGAAA GCCTTGGAAG TTTGGCCTCG CCTACTCCCT
ACA/Ner   ..-....... .......... .......... .-........ .......... .......... .......... .......... .......... ..........
AAT/Isr   ..-....... .......... .......... .-........ .......... .G........ .......... .......... .......... ..........
AAT/Tus   ..-....... .......... .......... .-...-.... ...-...... .......... .......... .......... .......... ..........
AAT/Pal   ..A....... .......... .......... .C........ .......... .......... .......... .......... .......... ..........
GG/Cag137 ..-....... .......... .......... .-........ .......... .......... .......... .......... .......... ..........
GB/Tbe4   ..-....... .......... .......... .-........ .......... .......... .......... .......... ........A. ..........
GM/Mar1   ..-....... .......... .......... .-........ .......... .......... .......... .......... ........A. ..........

                2110       2120       2130       2140       2150       2160       2170       2180       2190       2200
ACY/Epk4  GGGAGAAAAA ATAAGAGATG GAAGGAAAGA CTTTGCAATT GCGAAGTGGG TGTCGATATT GGGAAGTCGC CCCATATTCG TTGATACAGA TGGAAAAATG
ACA/Ner   .......... .......... .......... .......... .......... .......... .......... .......... .......... .......-..
AAT/Isr   .......... .......... .......... .......... .......... .......... .......... .......... .......... ..........
AAT/Tus   .......... .......... .......... .......... .......... .......... .......... .......... .......... .......-..
AAT/Pal   .......... .......... .......... .......... .......... .......... .......... .......... .......... .......-..
GG/Cag137 .......... .......... .......... .........A .......... ...T...... .....T.... .......... ......G... ..........
GB/Tbe4   .......... .......... .......... .........A .......... .......... .......... .......... ......G... ..........
GM/Mar1   .....G.... .......... .......... .........A .......... .......... .......... .......... ......G... ..........
```

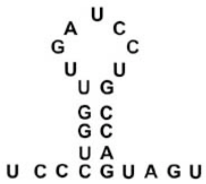


```
                2210       2220       2230       2240       2250       2260       2270       2280       2190       2300
ACY/Epk4  TTTATTATAA GATTCGGACC CGGTGCTCTG CGGACCCGGG ATGTTCCTCC GAACGAAAAA AGCTGCTCGC AGCTCCCTGG TTGATCCTGC CAGTAGTCAT
ACA/Ner   .......... .......... .......... .......... .......... .......... .......... .......... .......... ..........
AAT/Isr   .......... .......... .......... .......... .......... .......... .......... .......... .......... ..........
AAT/Tus   .......... .......... .......... .......... .......... .......... .......... .......... .......... ..........
AAT/Pal   .......... .......... .......... .......... .......... .......... .......... .......... .......... ..........
GG/Cag137 ..G....... .......... .......... .......... .......... .......... .......... .......... .......... ..........
GB/Tbe4   .......... .......... T......... .......... .......... .......... .......... .......... .......... ..........
GM/Mar1   .......... .......... T......... .......... .......... .......... .......... .......... .......... ..........
                                                                                      <---------------------------
```

```
                2310       2320       2330       2340       2350       2360       2370       2380       2390       2400
ACY/Epk4  ATGCTTGTCT CAAAGATTAA GCCATGCATG TCTAAGTGCA AGCCAAAATA AGGTGAAACC GCGAATGGCT CATTAAATCA GTTATGGTTC CTTAGATCGT
ACA/Ner   .......... .......... .......... .......... .......... .......... .......... .......... .......... ..........
AAT/Isr   .......... .......... .......... .......... .......... .......... .......... .......... .......... ..........
AAT/Tus   .......... .......... .......... .......... .......... .......... .......... .......... .......... ..........
AAT/Pal   .......... .......... .......... .......... .......... .......... .......... .......... .......... ..........
GG/Cag137 .......... .......... .......... .......... .......... .......... .......... .......... .......... ..........
GB/Tbe4   .......... .......... .......... .......... .......... .......... .......... .......... .......... ..........
GM/Mar1   .......... .......... .......... .......... .......... .......... .......... .......... .......... ..........
          -------------------------------------------------------------------------------------------------------------
                                                            18s

                2410       2420       2430       2440       2450       2460       2470       2480       2490       2500
ACY/Epk4  ACCACATGAC TTGGATAACT GTGGTAATTC TAGAGCTAAT ACATGCTGAA CTGAGTCCCG ACCAGAAATG GGAGGGATGC TTTTATTAGA TCAAAACCAA
ACA/Ner   .......... .......... .......... .......... .......... .......... .......... .......... .......... ..........
AAT/Isr   .......... .......... .......... .......... .......... .......... .......... .......... .......... ..........
AAT/Tus   .......... .......... .......... .......... .......... .......... .......... .......... .......... ..........
AAT/Pal   .......... .......... .......... .......... .......... .......... .......... .......... .......... ..........
GG/Cag137 .......... .......... .......... .......... .......... .......... .......... .......... .......... ..........
GB/Tbe4   .......... .......... .......... .......... .......A.. .......... .......... .......... .......... ..........
GM/Mar1   .......... .......... .......... .......... .......A.. .......... .......... .......... .......... ..........
          -------------------------------------------------------------------------------------------------------------
                                                            18s

                2510       2520       2530       2540       2550       2560       2570       2580       2590       2600
ACY/Epk4  TCGGCGTGGC TTGTCTGCGT CCGTTTGCTT TGGTGACTCT GGATAACTTT GTGCTGATCG CACGGTCTCC GTACCGGCGA CGCATCTTTC AAATGTCTGC
ACA/Ner   .......... .......... .......... .......... .......... .......... .......... .......... .......... ..........
AAT/Isr   .......... .......... .......... .......... .......... .......... .......... .......... .......... ..........
AAT/Tus   .......... .......... .......... .......... .......... .......... .......... .......... .......... ..........
AAT/Pal   .......... .......... .......... .......... .......... .......... .......... .......... .......... ..........
GG/Cag137 .......... .......... .......... .......... .......... .......... .......... .......... .......... ..........
GB/Tbe4   .......... .......... .......... .......... .......... .......... .......... .......... .......... ..........
GM/Mar1   .......... .......... .......... .......... .......... .......... .......... .......... .......... ..........
          -------------------------------------------------------------------------------------------------------------
                                                            18s

                2610       2620
ACY/Epk4  CTTATCAACT GTCGATGGTA
ACA/Ner   .......... ..........
AAT/Isr   .......... ..........
AAT/Tus   .......... ..........
AAT/Pal   .......... ..........
GG/Cag137 .......... ..........
GB/Tbe4   .......... ..........
GM/Mar1   .......... ..........
          ---------------------
```
