# Supplementary material for: The IGS-ETS in Bacillus (Insecta Phasmida): molecular characterization and the relevance of sex in ribosomal DNA evolution
Source: BMC Evol Biol. 2008 Oct 9;8:278. doi: 10.1186/1471-2148-8-278 (PMC2590618; doi:10.1186/1471-2148-8-278)
Supplement: Additional file 2 — Sequence alignment of the Bag530 sequences in Bacillus. Sequence alignment of the Bag530 sequences in Bacillus. Species acronyms as in Table 1. The "C" followed by a number indicates the number of clone sequenced. [file 1471-2148-8-278-S2.pdf]

Additional file 2 – Sequence alignment of the *Bag530* sequences in *Bacillus*

```
                        10         20         30         40         50         60         70         80         90        100
AAT/IsrC2      CTGCAGTCAT GTGGGCATAG AAAATGTTAA -ATTCCGCAG GCCTGAA-AA TTCTCCCGGC GATGGAGGCC TCCGCACCCC GGGAAATCCC -GGCGCCCGG
AAT/IsrC3      ......C... .....AG... .......... -........G .......-.. ....C..... ........G. ....C..... .......... -..C......
AAT/IsrC4      ......C... .....AG... .......... -........G .......-.. ....C..... ........C. ....C..... .......... -..C......
AAT/IsrC7      ......C... .....AG... .......... -........G .......-.. ....C..... ........C. ....C..... .......... -..C......
AAT/IsrC17     ......C..A .....AG... .......... -........G .......A.. ....C..... ........C. ....C....G .......... -..C......
AAT/IsrC19     ......C..A .....AG... .......... -........G .......A.. ....C..... ........C. ....C....G .......... -..C......
AAT/IsrC20     ......C..A .....AG... .......... -........G .......-.. ....C..... ........C. ....C..... .......... -..C......
AAT/IsrC22     ......C... .....AG... .......... -........G .......-.. ....C..... ........C. ....C..... .......... -..C......
AAT/IsrC23     ......C... .....GG... ......G... -........G .......A.- ....C..... ........C. ....C....G .......... -..C......
AAT/IsrC24     ......C... .....AG... .......... -........G .......-.. ....C..... ........C. ....C....G .......... -..C......
AAT/IsrC25     ......C... .....AG... .......... -........G .......-.. ....C..... ........C. ....C..... .......... -..C......
AAT/IsrC26     ......C... .....AG... .......... -........G .......-.. ....C..... C.......C. ....C..... .......... -..C......
AAT/PalC1      ......C... .....AG... G......... -........G .......-.. ....C..... ........C. ....C..... .......... -.TC......
AAT/PalC14     ......C... .....AG... G......... -........G .......-.. ....C..... ........C. ....C..... .......... -.TC......
AAT/PalC2      ......C... .....AG... G......... -........G .......-.. ....C..... ........C. ....C..... .......... -..C......
AAT/PalC8      ......C... .....AG... G......... -........G .......-.. ....C..... ........C. ....C..... .......... -..C......
AAT/PalC9      ......C... .....AG... G......... -........G .......-.. ....C..... ........C. ....C..... .......... -..C......
AAT/TusC1      ......C... .....AG... .......... -........G .......A.- ....C..... ........C. ....C..... .......... -..C......
AAT/TusC2      ......C... .....AG... G......... -........G .......-.. ....C..... ........C. ....C..... .......... -.TC......
AAT/TusC4      ......C... ....TAG.G. G...C..... -........G .......-.. ....C..... ........C. ....C..G.. .......... -..CC.....
AAT/TusC5      ......C... .....AG... .......... -........G .......T.- ....C..... ........C. ....C..G.. .......... -..C......
AAT/TusC7      ......C... .....TG... .......... -........G .......-.. ....C..... ........C. ....C..... .......... -.TC......
AAT/TusC8      ......C... .....AG... .......... -........G .......A.- ....C..... ........C. ....C..... .......... -..C......
AAT/TusC10     ......C... .....AG... .......... -........G .......-.. ....C..... ........C. ....C..... .......... -..C......
AAT/TusC17     ......C... .....AG... G......... -........G -......-.. ....C..... ........C. ....C..... .......... -.TC......
AAT/TusC20     ......C... .....AG... .......... -........G .......-.. ....C..... ........C. ....C..... .......... -..C......
ACA/NerC1      ......C... .....AG... G......... -........G .......A.- ....C..... ........C. ....C..... .......... -..C......
ACA/NerC2      ......C... .....AG..A .......... -........G .......A.- ....C..... ........C. ....C..... .......... -..C......
ACA/NerC3      ......C... .....AG... G......... -........G .......A.- ....C..... ........C. ....C..... .......... -.TC......
ACA/NerC4      ......C... .....AG... .......... -........G ..A....A.- ....C..... ........C. ....C..... .......... -..C......
ACA/NerC5      ......C... .....AG..A .......... -........G .......A.- ....C..... ........C. ....C..... .......... -.TC......
ACA/NerC6      ......C... .....AG... .......... -........G .......A.- ....C..... ........C. ....C..... .......... -.TC......
ACA/NerC7      ......C... .....AG... .......... -........G .......A.- ....C..... ........C. ....C..G.. .......... -..C......
ACA/NerC8      ......C... .....AG... .......... -........G .......A.- ....C..... ........C. ....C..... .......... -..C......
ACA/NerC10     ......C... .....AG..A .......... -........G .......A.- ....C..... ........C. ....C..... .......... -..C......
ACA/NerC15     ......C... .....AG... G......... -........G .......A.- ....C..... ........C. ....C..... .......... -.TC......
ACY/Epk3C4     ......C... .....AG... .......... -........G .......A.- ....C..... ........C. ....C..... .......... -..C......
ACY/Epk3C5     ......C... .....AG... .......... -........G .......-.. ....C..... ........C. ....C..... .......... -..C......
ACY/Epk3C6     ......C... .....AG... .......... -........G .......A.- ....C..... ........C. ....C..... .......... -..C......
ACY/Epk3C10    ......C... .....AG... .......... -........G .......-.. ....C..... ........C. ....C..... .......... -..C......
ACY/Epk4C1     ......C... .....AG... .......... -........G .......-.. ....C..... ........C. ....C..... .......... -..C......
ACY/Epk4C2     ......C... .....AG... .......... -T.......G .......-.. ....C..... ........C. ....C..... .......... -..C......
ACY/Epk4C4     ......C... .....AG... .......... -........G .......A.- ....C..... ........C. ....C..... .......... -..C......
ACY/Epk4C5     ......C... .....AG... .......... -........G .......A.- ....C..... ........C. ....C..... .......... -..C......
ACY/Epk4C8     ......C... .....AG... .......... -........G .......-.. ....C..... ........C. ....C..... C......... -..C......
ACY/Epk4C10    ......C... .....AG... .......... -........G .......-.. ....C..... ........C. ....C..... .......... -..C......
GM/Mar1C1      ......CA.. .....AG..T .......... -TAAA.AG.G ..-.A..A.. A...C..... ........C. ....C..... ......AA.. T..C......
GM/Mar1C2      ......CA.. .....AG..T .......... T.AA-.AG.G ..-.A..A.. A...C..... ........C. ....C..... ......AA.. T..C......
GM/Mar1C3      ......CA.. .....AG... .......... T.AA-.AG.G ..-.A..A.. ....C..... ........C. ..G.C..... ......AA.. T..C......
GM/Mar1C4      ......CA.. .....AG..T .......... T.AA-.AG.G ..-.A..A.. A...C..... ........C. ....C..... ......AA.. T..C......
GM/Mar1C5      ......CA.. .....AG..T .......... T.AA-.AG.G ..-.A..A.. A...C..... ........C. ...AC..... ......AA.. T..C......
GB/Tbe4C4      ......CA.. .....AG... .......... T.AAA.AG.G ..-.A..A.. ....C..... ........C. ..G.C..... ......AA.. T..C......
GB/Tbe4C5      ......CA.. ......G... .......... T.AAA.AG.G ..-.A..A.. ....C..... ........C. ..G.C..... ......AA.. T..C......
GB/Tbe4C8      ......CA.. .....AG... .......... T.AAA.AG.G ..-.A..A.. ....C..... ........C. ..G.C..... ......AA.. T..C......
GB/Tbe4C12     ......CA.. .....AG... .......... T.AAA.AG.G ..-.A..A.. ....C..... ........C. ..G.C..... ......AA.. T..C......
GB/Tbe4C13     ......CA.. .....AG... .......... T.AAA.AG.G ..-.A..A.. ....C..... ........C. ..G.C..... ......AA.. T..C......
GB/Tbe4C15     ......CA.. .....AG... .......... -TAAA.AG.G ..-.A..A.. ....C..... ........C. ..G.C..... ......AA.. T..C......
GG/Cag28C2     .......... T...CAG... .......... -.AAA.AG.G ..TA...-.. A...C..... ........C. ....C..A.. ......AA.. T..C....C.
GG/Cag137C2    ......N... T...TAG... .......... -TAAA.AG.G ..TA...-.. A...C..... ........C. ....C..A.. ......AA.. T..C....C.
GG/Cag137C5    ......C... T...CAG... .......... -.AAA.AG.G ..TA...-.. A...C..... ........C. ....C..A.. ......AA.. T..C....C.
GG/Cag139C8    ......C... T...CAT... .......... -.AAA.AG.G ..TA...-.. A...C..... ........C. ....C..A.. ......AA.. T..C....C.
GG/Cag139C11   ......C... T...CAG... ......G... -.AAA.AG.G ..TA...-.. A...C..... ........C. ....C..A.A ......AA.. T..C....C.
GG/Cag139C14   ......C... T...CAG... .......... -.AAA.AG.G ..TA...-.. A...C..... ........C. ....C..A.. ......AA.. T..C....C.
GG/Cag139C15   ......C... T...CAG... .......... -.AAA.AG.G ..-.AG.A.. A...C..... ........C. ....C..A.. ......AA.. T..C....C.
```

```
                          110        120        130        140        150        160        170        180        190        200
AAT/IsrC2       ACTTTGTGCG AGCCCCTCGC AGAATCCGAG CGAAACAGCC GAAATGAAAG GAGCGGGGAG CCAAAAAATT TCTCCCGGCG ATC-GAGGCC TCGATCGCCG
AAT/IsrC3       .......... .......... ..C....... .......... .......... .......... .......... .......... ...-...... ..........
AAT/IsrC4       .......... .......... ..C....... .......... .......... .......... .......... ...A...... ...-...... ..........
AAT/IsrC7       .......... .......... ..C.G..... .......... .......... .......... .......... .......... ...T...... ..........
AAT/IsrC17      .......... .......... ..C....... .......... .......... .......... .......... .......... ...-...... ..........
AAT/IsrC19      .......... .......... ..C.G..... .....A.... .......... .......... .......... .......... ...-...... ..........
AAT/IsrC20      .......... .........T ..C....... .......... .......... .......... .......... .......... ...-...... ..........
AAT/IsrC22      .......... .......... ..C....... .......... .......... .......... .......... .......... ...-...... ..........
AAT/IsrC23      .......... .......... ..C.G..... .......... .......... .......... .......... .......... ...-...... ..........
AAT/IsrC24      .......... .......... ..C.G..... .....A.... .......... .......... .......... .......... ...-...... ..........
AAT/IsrC25      .......... .......... ..C.G..... .......... .......... .......... .......... .......... ...-...... ..........
AAT/IsrC26      .......... .......... ..C....... .......... .......... .......... .......... .......... ...-...... ..........
AAT/PalC1       ........T. .......... ..C.G..... .......... .......... .......... .......... .......... ...-...... ..........
AAT/PalC14      ........T. .......... ..C.G..... .......... .......... .......... .......... .......... ...-...... ..........
AAT/PalC2       ........T. .......... ..C.G..... .......... .......... .......... .......... .......... ...-...... ..........
AAT/PalC8       ........T. .......... ..C.G..... .......... .......... .......... .......... .......... ...-...... ..........
AAT/PalC9       ........T. .......... ..C.G..... .......... .......... .......... .......... .......... ...-...... ..........
AAT/TusC1       ........T. .......... ..C.G..... .......... .......... .......... .......... .......... ...-...... ..........
AAT/TusC2       ........T. .......... ..C.G..... .......... .......... .......... .......... .......... ...-...... ..........
AAT/TusC4       ........T. .......... ..C.G..... .......... .......... .......... .......... .......... ...-...... ..........
AAT/TusC5       ........T. .......... ..C.G..... .......... .......... .......... .......... .......... ...-...... ..........
AAT/TusC7       ........T. .......... ..C.G..... .......... .......... .......... .......... .......... ...-...... ..........
AAT/TusC8       ........T. .......... ..C.G..... .......... .......... .......... .......... .......... ...-...... ..........
AAT/TusC10      ........T. .......... ..C.G..... .......... .......... .......... .......... .......... ...-...... ..........
AAT/TusC17      ........T. .......... ..C.G..... .......... .......... .......... .......... .......... ...-...... ..........
AAT/TusC20      ........T. .......... ..C.G..... .......... .......... .......... .......... .......... ...-...... ..........
ACA/NerC1       ........T. .......... ..C.G..... .......... .......... .......... .......... .......... ...-...... ..........
ACA/NerC2       ........T. .......... ..C.G..... .......... .......... .......... .......... .......... ...-...... ..........
ACA/NerC3       ........T. .......... ..C.G..... .......... .......... .......... .......... .......... ...-...... ..........
ACA/NerC4       ........T. .......... ..C.G..... .......... .......... .......... .......... .......... ...-...... ..........
ACA/NerC5       ........T. .......... ..C.G..... .......... .......... .......... .......... .......... ...-...... ..........
ACA/NerC6       ........T. .......... ..C.G..... .......... .......... .......... .......... .......... ...-...... ..........
ACA/NerC7       ........T. .......... ..C.G..... .......... .......... .......... .......... .......... ...-...... ..........
ACA/NerC8       ........T. .......... ..C.G..... .......... .......... .......... .......... .......... ...-...... ..........
ACA/NerC10      ........T. .......... ..C.G..... .......... .......... .......... .......... .......... ...-...... ..........
ACA/NerC15      ........T. .......... ..C.G..... .......... .......... .......... .......... .......... ...-...... ..........
ACY/Epk3C4      .......... .......... .......... .......... .......... .......... .......... .......... ...-...... ..........
ACY/Epk3C5      .......... .......... .......... .......... .......... .......... .......... .......... ...-...... ..........
ACY/Epk3C6      .......... .......... .......... .......... .......... .......... .......... .......... ...-...... ..........
ACY/Epk3C10     .......... .......... .......... .......... .......... .......... .......... .......... ...-...... ..........
ACY/Epk4C1      .......... .......... ..C.G..... .......... .......... .......... .......... .......... ...-...... ..........
ACY/Epk4C2      .......... .......... ..C....... .......... .......... .......... .......... .......... ...-...... ..........
ACY/Epk4C4      .......... .......... .......... .......... .......... .......... .......... .......... ...-...... ..........
ACY/Epk4C5      .......... .......... .......... .......... .......... .......... .......... .......... ...-...... ..........
ACY/Epk4C8      .......... .........A ..C....... .......... .......... .......... .......... .......... ...-...... ..........
ACY/Epk4C10     .......... .......... ..C....... .......... .......... .......... .......G.. .......... ...-...... ..........
GM/Mar1C1       ........T. C......... ..C.G..A.. .......... .........C ........CC .T.G....A. .......... ...C...... ..........
GM/Mar1C2       ........T. C....T.... ..C.G..A.. .......... .........C ........CC .T.G....A. .......... ...-...... ..........
GM/Mar1C3       ........T. C......... ..C.G..A.. .......... A........C ........CC .T.G....A. .......... ...-...... ..T.......
GM/Mar1C4       ........T. C......... ..C.G..A.. .......... .........C ........CC .T.G....A. .......... ...-...... ..........
GM/Mar1C5       ........T. C......... ..C.G..A.. .......... .........C ........CC .T.G....A. .......... ...-...... ..........
GB/Tbe4C4       ........T. C......... ..C.G..A.. .......... .........C ........CC .T.G....A. .......... ...-...... ..........
GB/Tbe4C5       ........T. C......... ..C.G..A.. .......... .........C ........CC .T.G....A. .......... ...-...... ..........
GB/Tbe4C8       ........T. C......... ..C.G..A.. .......... .........C ........CC .T.G....A. .......... ...-...... ..........
GB/Tbe4C12      ........T. C......... ..C.G..A.. .......... .........C ........CC .T.G....A. .......... ...-...... ..........
GB/Tbe4C13      ........T. C......... ..C.G..A.. .......... .........C ........CC .T.G....A. .......... ...-...... ..........
GB/Tbe4C15      ........T. C......... ..C.G..A.. .......... .........C ........CC .T.G....A. .......... ...-...... ..........
GG/Cag28C2      ........T. C......... ..C.CA.... .......... .......... ........CC .TTG....A. .......... ...-...... ..........
GG/Cag137C2     ........T. C......... ..C.CA...T .......... .......... ........CC .TTG....A. .......... ...-...... ..........
GG/Cag137C5     ........T. C..A...... ..C.CA.... .......... .......... ........CC .TTG....A. .......... ...-...... ..........
GG/Cag139C8     ........T. C......... ..C.CA.... .......... .......... ........CC .TTG....A. .......... ...-...... ..........
GG/Cag139C11    ........T. C..A...... ..C.CA.... .......... .......... ........CC .TTG....A. .......... ...-...... ..........
GG/Cag139C14    ........T. C......... ..C.CA.... .......... .......... ........CC .TTG....A. .......... ...-.T.... ..........
GG/Cag139C15    ........T. C......... ..C.CA.... .......... ..T..T.... ........CC .TTG....A. .......... ...-...... ..........
```

```
                      210        220        230        240        250        260        270        280        290        300
AAT/IsrC2      GGGAAATCAC CTTCGCCCGG ACTTGCCGAG CACCCAACGA GACTGTCAAT TTGGGTTATA TAGGGGG--T AGTGG-TGTC TCTAGA-GGT G-AAAAAAAA
AAT/IsrC3      .......... .......... G-........ .......... .......... ...T...... .......--. .....-.... ......A..G .G.......T
AAT/IsrC4      .......... .......... GT........ .......... .......... ...T...... .......--. .....-.... ......AC.G .G.......T
AAT/IsrC7      .......... .......... G......... .......... .......... .......... .......--. .....-.... ......--.G TG.......T
AAT/IsrC17     .......... .......... G......... .......... .......... .......... .......--. .....-.... ......A..G .-......TT
AAT/IsrC19     .......... .......... G......... ..---..... .......... .......... .......--. .....-.... ......A..G .-......TT
AAT/IsrC20     .......... .......... G......... .......... .......... .......... .......--. .....-.... ......-... .-.......T
AAT/IsrC22     .........T .......... G......... .......... .......... .......... .......--. .....-.... ......A..G .-.......T
AAT/IsrC23     .......... .......... G......... .......... .......... .......... .......--. .....-.... ......A..G .-.......T
AAT/IsrC24     .......... .......... G......... ..---..... .......... .......... .......--. .....-.... ......A..G .-......TT
AAT/IsrC25     .......... .......... G......... .......... .......... .......... .......--. .....-.... ......--.G TG.......T
AAT/IsrC26     .......... .......... G......... .......... .......... .......... .......--. .....-.... ......A-.G .G......TT
AAT/PalC1      .......... .......... G......... .......... .......... .......... .......--. .....-.... ......A-.G .G....TTTT
AAT/PalC14     .......... ....G..... G......... .......... .......... .......... .......--. .....-.... ......A-.G .G.....TTT
AAT/PalC2      .......... .......... G........T .......... .......... .......... .......GG. .....-.T.. ......A-.G .G.......T
AAT/PalC8      .......... .......... G......... .......... .......... .......... .......--. .....-.T.. ......A..G .-.......T
AAT/PalC9      .......... .......... G........T .......... .......... .......... .......--. .....-.... ......A..G .-......TT
AAT/TusC1      .......... .......... G......... .......... .......... .......... .......--. .....-.... ......A..G .-.....TTT
AAT/TusC2      .......... .......... G......... .......... .......... .......... .......--. .....-.... ......A..G .-.....TTT
AAT/TusC4      .......... .......... G......... .......... .......... .......... .......--. .....-.... ......A..G .-.....TTT
AAT/TusC5      .......... .......... G......... .......... .......... .......... .......--. .....-.... ......A..G .-.....TTT
AAT/TusC7      .......... .......... G......... .......... .......... .......... .......--. .....-.... ......A..G .-.....TTT
AAT/TusC8      .......... .......... G......... .......... .......... .......... .......--. .....-.... ......A..G .-.....TTT
AAT/TusC10     .......... .......... G......... .......... .......... .......... .......--. .....-.... ......A-.G .G......TT
AAT/TusC17     .......... .......... G......... .......... .......... .......... .......--. .....-.... ......A..G .-.....TTT
AAT/TusC20     .......... .......... G......... .......... .......... .......... .......--. .....-.... ......A..G .-.....TTT
ACA/NerC1      .......... .......... G........T .......... .......... .......... .......--. .....-..-- ......A..G .-.....TTT
ACA/NerC2      G......... .......... G........T .......... .......... .......... .......--. .....-..-- ......A..G .-.......T
ACA/NerC3      .......... .......... G......... .......... .......... .......... .......--. .....-..-- ......A..G .-.....TTT
ACA/NerC4      .......... .......... G........T .......... .......... .......... .......--- .....-..-- ......A..G .-......TT
ACA/NerC5      .......... .......... G........T .......... .......... .......... .......--G .....-..-- ......A..G .-.......T
ACA/NerC6      .......... .......... G........T .......... .......... .......... .......--. .....-..-- ......A..G .-.....TTT
ACA/NerC7      .......... .......... G......... .......... .......... .......... .......--. .....-..-- ......A..G .-......TT
ACA/NerC8      .......... .......... G......... .......... .......... .......... .......--. .....-..-- ......A..G .-.....TTT
ACA/NerC10     .......... .......... G........T .......... .......... .......... .......--. .....-..-- ......A..G .-.....TTT
ACA/NerC15     .......... .......... G......... .......... .......... .......... .......--. .....-..-- ......A..G .-.....TTT
ACY/Epk3C4     .......... .......... G......... .......... .......... .......... .......--. .....-.... ......A..G --.......T
ACY/Epk3C5     .......... .......... G......... .......... .......... .......... .......--. .....-.... ......A..- -G.......T
ACY/Epk3C6     .......... .......... G......... .......... .......... .......... .......--. .....-.... ......A..G A-......TT
ACY/Epk3C10    .......... .......... .......... .......... .......... .......... .......--. .....-.... ......-... .-........
ACY/Epk4C1     .......... .......... G......... .......... .......... .......... .......--. .....-.... ......A..- .-.......T
ACY/Epk4C2     .......... .......... G......... .......... .......... .......... .......--. .....-.... ......A-.G .G......TT
ACY/Epk4C4     .......... .......... G......... .......... .......... .......... .......--. .....-.... ......A..G A-......TT
ACY/Epk4C5     .......... .......... G......... .......... .......... .......... .......--. .....-.... ......A..G A-......TT
ACY/Epk4C8     .......... .......... G......... .......... .......... .......... .......--. .....-.... ......-... .-.......T
ACY/Epk4C10    .......... .......... G......... .......... .......... .......... .......--. .....-.... ......A..G .-......TT
GM/Mar1C1      .......... GGG....... G.....T.TT .C..TGC... .......... .......... .......--. .....-.... ......A..G -A.....-TT
GM/Mar1C2      .......... GGG....... G.....T.T. .C..TGT... .......... .......... .......--. .....-.... ......A..G --.....TTT
GM/Mar1C3      .......... GG........ G.....T.T. .C..TGT... .......... .......... .......--. .....-.... ......A..G --.....TTT
GM/Mar1C4      .......... GGG....... G.....T.TT .C..TGC... .......... .......... .......--. .....-.... ......A..G --......TT
GM/Mar1C5      .......... GGG....... G.....T.T. GC..TGT... .......... .......... .......--. .....-.... ......A..G --......TT
GB/Tbe4C4      .......... GGG....... G.....T.T. .C..TGT... .......... ....T..... .......--. .....-.... ......A..G .-.....TTT
GB/Tbe4C5      .......... GGG....... G.....T.T. .C..TGT... .......... .......... .......--. .....-.... ......A..G .-.....TTT
GB/Tbe4C8      .......... GGG....... G.....T.T. .C..TGT... .......... .......... .......--. .....-.... ......A..G .-.....TTT
GB/Tbe4C12     .......... GGG....... G.....T.T. .C..TGT... .......... .......... .......--. .....-.... ......A..G .-.....TTT
GB/Tbe4C13     .......... GGG....... G.....T.T. .C..TGT... .......... .......... .......--. .....-.... ......A..G .-.....TTT
GB/Tbe4C15     .......... GGG....... G.....T.T. .C..TGT... .......... .......... .......--. .....-.... ......A..G -G.....TTT
GG/Cag28C2     .......... GGG.C..... G.....T.C. .C..T.T..G .......... .......... .......--. .....-..C. ......A-.G .A......TT
GG/Cag137C2    .......... GGG.C..... G.....T.C. .C..T.T..G .......... .......... .......--. .....-..C. ......A--G .G.....TTT
GG/Cag137C5    .......... GGG.C..... G.....T.CG .C..T.T..G .......... .......... .......--. .....G..C. ......A-.G .-......TT
GG/Cag139C8    .......... GGG.C..... G.....T.C. .C..T.T..G .......... .......... .......--. .....-..C. ......A-.G .A....TTTT
GG/Cag139C11   .......... GGG.C..... G.....T.C. .C..T.T..G .......... .......... .......--. .....-..C. ......A--G .G.......T
GG/Cag139C14   .......... GGG.CT.... G.....T.C. .C..T.T..G .......... .......... .......--. .....-..C. ......A-.G .-......TT
GG/Cag139C15   .......... GGG.C..... G.....T.C. .C..T.T..G .......... .......... .......--. .....-..C. ......A..G -A......TT
```

```
                  310        320        330        340        350        360        370        380        390        400
AAT/IsrC2       TTTTTCAAGT ATTGCGCCC- GGGGACAAGA GAAATCTCTC GGCGATCGAG GCCTCCATAG GCCGGTAAAA -TTCCGGCGC CCGGACTATG TGTCTTCCCT
AAT/IsrC3       .......... .........- .......... .......... ..T....... .......... .......... -......... .......... ..........
AAT/IsrC4       .......... .......-.C .......... .......... ..T....... .......... .......... -......... .......... ..........
AAT/IsrC7       .......... .......-.C .......... .......... .......... .......... .......... -......... .......... ..........
AAT/IsrC17      .......... .........- .......... .......... .......... .......... .......... -......... .......... ..........
AAT/IsrC19      .......... .........- .......... .......... .......... .......... .......... -......... .......... ..........
AAT/IsrC20      .......... .........- .......... .......... .......... .......... .......... -......... .......... ..........
AAT/IsrC22      .......... .........- .......... .......... .......... .......... .......... -......... .......... ..........
AAT/IsrC23      .......... .........- .......... .......... .......... .......... .......... -......... ....N..... ..........
AAT/IsrC24      .......... .........- .......... .......... .......... .......... .......... -......... .......... ..........
AAT/IsrC25      .......... .......G.C .......... .......... .......... .......... .......... -......... .....A.... ..........
AAT/IsrC26      .......... .........- .......... .......... .......... .......... .....G.... A......... .......... ..........
AAT/PalC1       .......... .........G ..A....T.. .......... .......... .......... .......... -......... .......... ..........
AAT/PalC14      .......... .........G ..A....T.. .......... .......... .......... .......... -......... .......... ..........
AAT/PalC2       .......... .........- .......... .......... .......... .......... .......... -......... .......... ..........
AAT/PalC8       .......... .........- .......... .......... .......... .......... .......... -......... .......... ..........
AAT/PalC9       .......... .........- .....G.... .......... .......... .......... .......... -......... .......... ..........
AAT/TusC1       .......... .........- .......... .......... .......... .......... .......... -......... .......... ..........
AAT/TusC2       .......... .........- .......T.. .......... .......... .......... .......... -......... .......... ..........
AAT/TusC4       .......... .........- .......... .......... .......... .......... .......... -......... .......... ..........
AAT/TusC5       .......... .........- .......T.. .......... .......... .......... .......... -......... .......... ..........
AAT/TusC7       .......... .........- .......T.. .......... .....C.... .......... .......... -......... .......... ..........
AAT/TusC8       .......... .........- .......... .......... .......... .......... .......... -......... .......... ..........
AAT/TusC10      .......... .........- .......T.. .......... .......... .......... .......... -......... .......... ..........
AAT/TusC17      .......... .........- .......T.. .......... .......... .......... .......... -......... .......... ..........
AAT/TusC20      .......... .........- .......T.. .......... .......... .......... .......... -......... .......... ..........
ACA/NerC1       .......... .........- .......T.. .......... .......... .......... .......... -......... .......... ..........
ACA/NerC2       .......... .........- .......... .......... .......... .......... .......... -......... .......... ..........
ACA/NerC3       .......... .........- .......T.. .......... .......... .......... .......... -......... .......... ..........
ACA/NerC4       .......... .........- .....G.... .......... .......... .......... .......... -......... .......... ..........
ACA/NerC5       .......... .........- .......... .......... .......... .......... .......... -......... .......... ..........
ACA/NerC6       .......... .........- .......T.. .......... .......... .......... .......... -......... .......... ..........
ACA/NerC7       .......... .........- .....G.... .......... .......... .......... .......... -......... .......... ..........
ACA/NerC8       .......... .........- .......T.. .......... .......... .......... .......... -......... .......... ..........
ACA/NerC10      .......... .........- .......T.. .......... .......... .......... .......... -......... .......... ..........
ACA/NerC15      .......... .........- .......T.. .......... .......... .......... .......... -......... .......... ..........
ACY/Epk3C4      .......... .........- .......... .......... .......... .......... .......... -......... .......... ..........
ACY/Epk3C5      .......... .........- .......... .......... .......... .......... .......... -......... .......... ..........
ACY/Epk3C6      ....-..... .........- .......... .......... .......... .......... .......... -......... .......... ..........
ACY/Epk3C10     .......... .........- .......... .......... .......... .......... .......... -......... .......... ..........
ACY/Epk4C1      .......... .........- .......... .......... .......... .......... .......... -......... .......... ..........
ACY/Epk4C2      .......... .........- .......... .......... .......... .......... .......... -......... .......... ..........
ACY/Epk4C4      ....-..... .........- .......... .......... .......... .......... .......... -......... .......... ..........
ACY/Epk4C5      ....-..... .........- .......... .......... .......... .......... .......... -......... .......... ..........
ACY/Epk4C8      ........A. .........- .......... .......... .......... .......... .......... -......... .......... ..........
ACY/Epk4C10     .......... .........- .......... .......... .......... .......... .......... -......... .......... ..........
GM/Mar1C1       .......... .........- ......C... .......... ......A... .......C.T .......... -..T...... .......... ..........
GM/Mar1C2       .......... .........- ......C... .......... ......A... .......C.T .......... -..T...... .......... ..........
GM/Mar1C3       .......... ......G..- ......C... .......... .......... .......C.T .......... -..T...... .......... ..........
GM/Mar1C4       .......... .........- ......C... .......... ......A... .......C.T .......... -..T...... .......... ..........
GM/Mar1C5       .......... .........- ......C... .......... ......A... .......C.T .......... -..T...... .......... ..........
GB/Tbe4C4       .......... .....C...- C.....G... .......... ......G... .......C.T .......... -..T...... ...A.C.... ..........
GB/Tbe4C5       .......... .........- ......G... .......... ......G... .......C.T .......... -..T...... .......... ..........
GB/Tbe4C8       .......... .........- ......G... .......... ......G... .......C.T .........- -..T...... .......... ..........
GB/Tbe4C12      .......... .........- ......G... .......... ......G... .....A.C.T .......... -..T...... .......... ..........
GB/Tbe4C13      .......... .........- ......G... .......... ......G... .....A.C.T .......... -..T...... .......... ..........
GB/Tbe4C15      .......... .........- ......G... .......... ......G... .......C.T .......... -..T...... .......... ..........
GG/Cag28C2      ....C..-.C .........G ..-...G... .......... ......A... .......C.T .......... -..T...... .......... .TC.......
GG/Cag137C2     ......C..C .......-.C ......G... .......... .......... .......C.T .......... -..T...... ........-. .TC.......
GG/Cag137C5     ......C..C .........- ......G... .......... .......... .......C.T .......... -..T...... .......... .TC.......
GG/Cag139C8     ....C..-.C .........G ..-...G... .......... .......... .......C.T .......... -..T...... .......... .TC.......
GG/Cag139C11    ......C..C .......-.C ......G... .......... .......... .......C.T .......... -..T...... .......... .TC.......
GG/Cag139C14    ......C..C .........- ......G... .......... .......... .......C.T .......... -..T...... .......... .TC.......
GG/Cag139C15    ....-.C..C .........- ......G... .......... .......... .......C.T .......... -..T...... .......... .TC.......
```

```
                        410        420        430        440        450        460        470        480        490        500
AAT/IsrC2      T-CTAGACAG GCAAATGGGA GTTGAAAAAT GTCATACCCC GAGACATGAA AAATCT-CCC GGCGGCCGAG GCCTCCACAC CCCGGGACGC TC-CTGGCGC
AAT/IsrC3      .-........ .......... .......... .......... .......... ......-... .......... .......... .......... ..-.......
AAT/IsrC4      .-........ .......... .......... .......... .......... ......-... .A........ .......... .......... ..-.......
AAT/IsrC7      .-........ .......... .......... .......... .......... ......-... .......... .......... .......... ..-.......
AAT/IsrC17     .-........ .......... .......... .......... .......... ......T... .......... .......... .......... ..-.......
AAT/IsrC19     .-........ .......... .......... .......... .......... ......T... .......... .......... .......... ..-.......
AAT/IsrC20     .-........ .......... .......... .......... .......... ......A... .......... .......... .......... ..-.......
AAT/IsrC22     .-........ .......... .......... .......... .......... ......-... .......... .......... .......... ..-.......
AAT/IsrC23     .-........ .......... .......... .......... .......... ......-... .......... .......... .......... ..-.......
AAT/IsrC24     .-........ .......... .......... .......... .......... ......T... .......... .......... .......... ..-.......
AAT/IsrC25     .-........ .......... .......... .......... .......... ......T... .......... .......... .......... ..-.......
AAT/IsrC26     .-........ .......... .......... .......... .......... ......-... .CG.C..... .......... .......... ..-.......
AAT/PalC1      .-........ .......... .......... .......... ..A....... ......-... .....T.... .......... .......... ..-.......
AAT/PalC14     .-........ .......... .......... .......... ..A....... ......-... .A...T.... .......... .......... ..-.......
AAT/PalC2      .-........ .......... .......... .......... .......... ......-... .....T.... .......... .......... ..-.......
AAT/PalC8      .-........ .......... .......... .......... .......... ......-... .....T.... .......... .......... ..-.......
AAT/PalC9      .-........ .......... .......... .......... .......... ......-... .....T.... .......... .......... .T-.......
AAT/TusC1      .-........ ..T....... .......... .......... .......... ......-... .....T.... .......... .......... ..-.......
AAT/TusC2      .-........ .......... .......... .......... .......... ......-... .....T.... .......... .........- ..-.......
AAT/TusC4      .-........ .......... .......... .......... .........T ......-... .....T.... .....A.A.. ........G. ..-....GC.
AAT/TusC5      .-........ .GC....... .......... .......... .......... ......-... .....T.... .......... .........T ..-.......
AAT/TusC7      .-........ ..T....... .......... .......... .......... ....-.-... .....T.... .......... .........- ..-.......
AAT/TusC8      .-........ ..T....... .......... .......... .......... ......-... .....T.... .......... .......... ..-.......
AAT/TusC10     .-........ .......... .......... .......... .......... ......-... .....T.... .......... .......... ..-.......
AAT/TusC17     .-........ .......... .......... .......... .......... ......-... .....T.... .......... .......... ..-.......
AAT/TusC20     .-........ .......... .......... .......... .......... ......-... .....T.... .......... .........- ..-.......
ACA/NerC1      .-........ .GC....... .......... .......... .......... ......-... .....T.... .......... .........T ..-.......
ACA/NerC2      .-........ .......... .......... .......... .......... ......-... .....T.... .......... .......... ..-.......
ACA/NerC3      .-........ .......... .......... .......... .......... ......-... .....T.... .......... .......... ..-.......
ACA/NerC4      .-........ .......... .......... .......... .......... ......-... .....T.... .......... .......... .T-.......
ACA/NerC5      .-........ .......... .......... .......... .......... ......-... .....T.... .......... .......... ..-.......
ACA/NerC6      .-........ .......... .......... .......... .......... ......-... .....T.... .......... .........- ..-.......
ACA/NerC7      .-........ .......... .......... .......... .......... ......-... .....T.... .......... .......... .T-.......
ACA/NerC8      .-........ .GC....... .......... .......... .......... ......-... .....T.... .......... .........T ..-.......
ACA/NerC10     .-........ .......... .......... .......... .......... ......-... .....T.... .......... .........- ..-.......
ACA/NerC15     .-........ .......... .......... .......... .......... ......-... .....T.... .......... .......... ..-.......
ACY/Epk3C4     .-........ .......... .......... .......... .......... ......-... .......... .......... .......... ..-.......
ACY/Epk3C5     .-........ .......... .......... .......... .......... ......-... .......... .......... .......... ..-.......
ACY/Epk3C6     .-........ .......... .......... .......... .......... ......-... .......... .......... .......... ..-.......
ACY/Epk3C10    .-........ .......... .......... .......... .......... ......-... .......... .......... .......... ..-.......
ACY/Epk4C1     .-........ .......... .......... .......... .......... ......-... .......... .......... .......... ..-.......
ACY/Epk4C2     .-........ .......... .......... .......... .......... ......-... .......... .......... .......... ..-.......
ACY/Epk4C4     .-........ .......... .......... .......... .......... ......-... .......... .......... .......... ..-.......
ACY/Epk4C5     .-........ .......... .......... .......... .......... ......-... .......... .......... .......... ..-.......
ACY/Epk4C8     .-........ .......... .......... .......... .......... ......-... .......... .......... .......... ..-.......
ACY/Epk4C10    .-........ .......... .......... .......... .......... ......-... .......... .......... .......... ..-.......
GM/Mar1C1      .-........ .........T .......... .......... ....G..... ......-... ....ATA... .......... .....C.A.G ..-.......
GM/Mar1C2      .-........ .........T .......... .......... ....G..... ......-... ....AT.... .......... .....C.A.G ..-.......
GM/Mar1C3      .-........ .........T .......... .......... ....G..... ......-... ....AT.... .......... .....C.A.G ..-.......
GM/Mar1C4      .-........ .........T .......... .......... ....G..... ......-... ....ATA... .......... .....C.A.G ..-.......
GM/Mar1C5      .-........ .........T .......... .......... ....G..... ......-... ....AT.... .......... .....C.A.G ..-.......
GB/Tbe4C4      .-........ .........T .......... .......... ....G..... ......-... ....AT.... .......... .....C.A.G ..-.......
GB/Tbe4C5      .G........ .........T .......... .......... ....G..... ......-... ....AT.... .......... .....C.A.G ..-.......
GB/Tbe4C8      .-........ .........T .......... .......... ....G..... ......-... ....AT.... .......... .....C.A.G ..-.......
GB/Tbe4C12     .-........ .........T .......... .......... ....G..... ......-... ....AT.... .......... .....C.A.G ..-.......
GB/Tbe4C13     .-........ ......T..G .......... .......... ....G.T... ......-... ....AT.... .......... .....C.A.G ..-.......
GB/Tbe4C15     .-........ .........T .......... .........- ....G..... ......-... ....AT.... .......... .....C.A.G ..-.......
GG/Cag28C2     .-...T.... .......... .......... .......... .......... ......-... ....AT.... .......... .......A.G ..-.......
GG/Cag137C2    .-...T.... .......... .......... .......... .......... ......-... ....AT.... .......... .......A.G ..A.......
GG/Cag137C5    .-...T.... .......... .......... .......... .......... ......-... ....AT.... .......... .......A.G ..-.......
GG/Cag139C8    .-...T.... .......... .......... .......... .......... ......-... ....AT.... .......... .......A.G ..-.......
GG/Cag139C11   .-...T.... .......... .......... .......... .......... ......-... ....AT.... .......... .......A.G ..-.......
GG/Cag139C14   .-...T.... .......... .......... .......... .......... ......-... ....AT.... .......... .......A.G ..-.......
GG/Cag139C15   .-...T.... .......... .......... .......... .......... ......-... ....AT.... .......... .......A.G ..-.......
```

```
                        510         520        530
AAT/IsrC2      ACGGA-CTTT GTGTCGGCAC CTC-GCTGCA G
AAT/IsrC3      .....-.... .......... ...-...... .
AAT/IsrC4      .....-.... .......... ...-A..... .
AAT/IsrC7      .....-.... .......... ...-...... .
AAT/IsrC17     .....-.... ........N. ...-...... .
AAT/IsrC19     .....-.... .......... ...-...... .
AAT/IsrC20     .....-.... .......... ..G-...... .
AAT/IsrC22     .G...-.... .......... ...-...... .
AAT/IsrC23     .....-.... .......... ...-...... .
AAT/IsrC24     .....-.... .......... ...-...... .
AAT/IsrC25     .....-.... .......... ...-...... .
AAT/IsrC26     .....-.... .......... ...-...... .
AAT/PalC1      .....-.... .......... ...-A..... .
AAT/PalC14     .....-.... .......... ...-A..... .
AAT/PalC2      .....-.... .......... ...-...... .
AAT/PalC8      .....-.... .......... ...-...... .
AAT/PalC9      .....-.... .......... ...-...... .
AAT/TusC1      .....-.... .......... ...-...... .
AAT/TusC2      .....-.... .......... ...-...... .
AAT/TusC4      .....-.... .......... ...-...... .
AAT/TusC5      .....-.... .......... ...-...... .
AAT/TusC7      .....-.... .......... ...-...... .
AAT/TusC8      .....-.... .......... ...-...... .
AAT/TusC10     .....-.... .......... ...-...... .
AAT/TusC17     .....-.... .......... ...-...... .
AAT/TusC20     ....G-A... .......... ...-...... .
ACA/NerC1      .....-.... .......... ...-...... .
ACA/NerC2      .....-.... .......... ...-...... .
ACA/NerC3      .....-.... .......... ...-...... .
ACA/NerC4      .....-.... .......... ...-...... .
ACA/NerC5      .....-.... .......... ...-...... .
ACA/NerC6      ....G-A... .......... ...-...... .
ACA/NerC7      .....-.... .......... ...-...... .
ACA/NerC8      .....-.... .......... ...-...... .
ACA/NerC10     ....G-A... .......... ...-...... .
ACA/NerC15     .....-.... .......... ...-...... .
ACY/Epk3C4     .....-.... .......... ...-...... .
ACY/Epk3C5     .....-.... .........T .A.-...... .
ACY/Epk3C6     .....-.... .......... ...-...... .
ACY/Epk3C10    .....-.... .......... ...-...... .
ACY/Epk4C1     .....-.... .......... ...-...... .
ACY/Epk4C2     .....-.... .......... ...-...... .
ACY/Epk4C4     .....-.... .......... ...-...... .
ACY/Epk4C5     .....-.... .......... ...-...... .
ACY/Epk4C8     .....-.... .......... ...-...... .
ACY/Epk4C10    .....-.... .......... ...-...... .
GM/Mar1C1      .....-.... .....T.... ...-...... .
GM/Mar1C2      .....-.... .....T.... ...-...... .
GM/Mar1C3      .....-.... .....T.... ...-...... .
GM/Mar1C4      .....-.... .....T.... ...-...... .
GM/Mar1C5      .....-.... ....TT.... ...-...... .
GB/Tbe4C4      .....G.... .....T.... T..AG..... .
GB/Tbe4C5      .....-.... .....T.... T..A...... .
GB/Tbe4C8      .....-.... .....T.... T..-...... .
GB/Tbe4C12     .....-.... .....T.... T..-...... .
GB/Tbe4C13     .....-.... .....T.... T..-...... .
GB/Tbe4C15     .....-.... .....T.... T..-...... .
GG/Cag28C2     .....-.... .....----- ------.... .
GG/Cag137C2    .....-.... .....----- ------.... .
GG/Cag137C5    .....A.... .....----- ------.... .
GG/Cag139C8    .....-.... .....----- ------.... .
GG/Cag139C11   .....-.... .....----- ------.... .
GG/Cag139C14   .....-.... .....----- ------.... .
GG/Cag139C15   .....-.... .....----- ------.... .
```
