# Supplementary figures and images for: The IGS-ETS in Bacillus (Insecta Phasmida): molecular characterization and the relevance of sex in ribosomal DNA evolution
Source: BMC Evol Biol. 2008 Oct 9;8:278. doi: 10.1186/1471-2148-8-278 (PMC2590618; doi:10.1186/1471-2148-8-278)

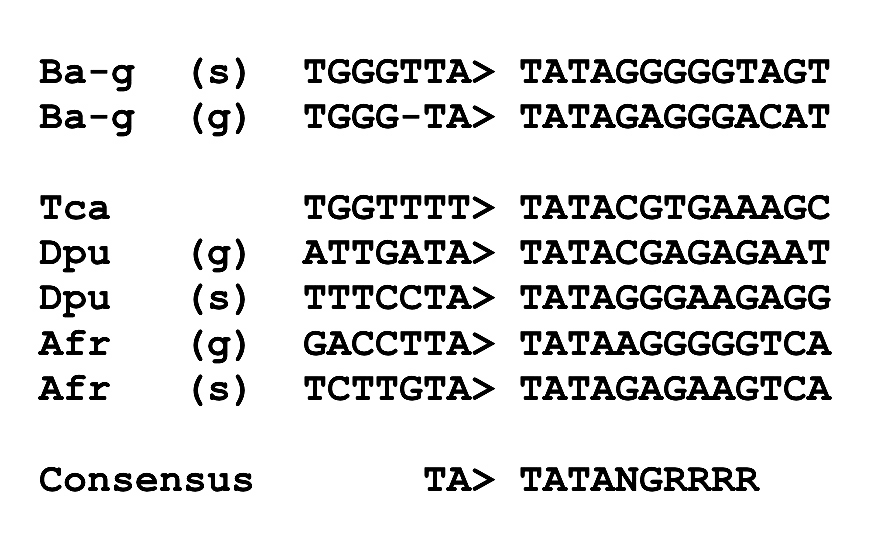

Supplement: Additional file 3 — Putative promoter sequences comparisons. Putative promoter sequence of B. atticus and B. grandii (Ba-g). Promoter sequences of Triops cancriformis (Tca), Daphnia pulex (Dpu) and Artemia franciscana (Afr) were also reported for comparison. The first nucleotide of the promoter sequence is indicated by (>) and seven bp of the upstream sequence are reported; (g) and (s) indicate gene and spacer promoters respectively. Consensus sequence of regions surrounding rDNA tsp of several arthropods is reported, as described by Crease (1993). [file 1471-2148-8-278-S3.jpeg]

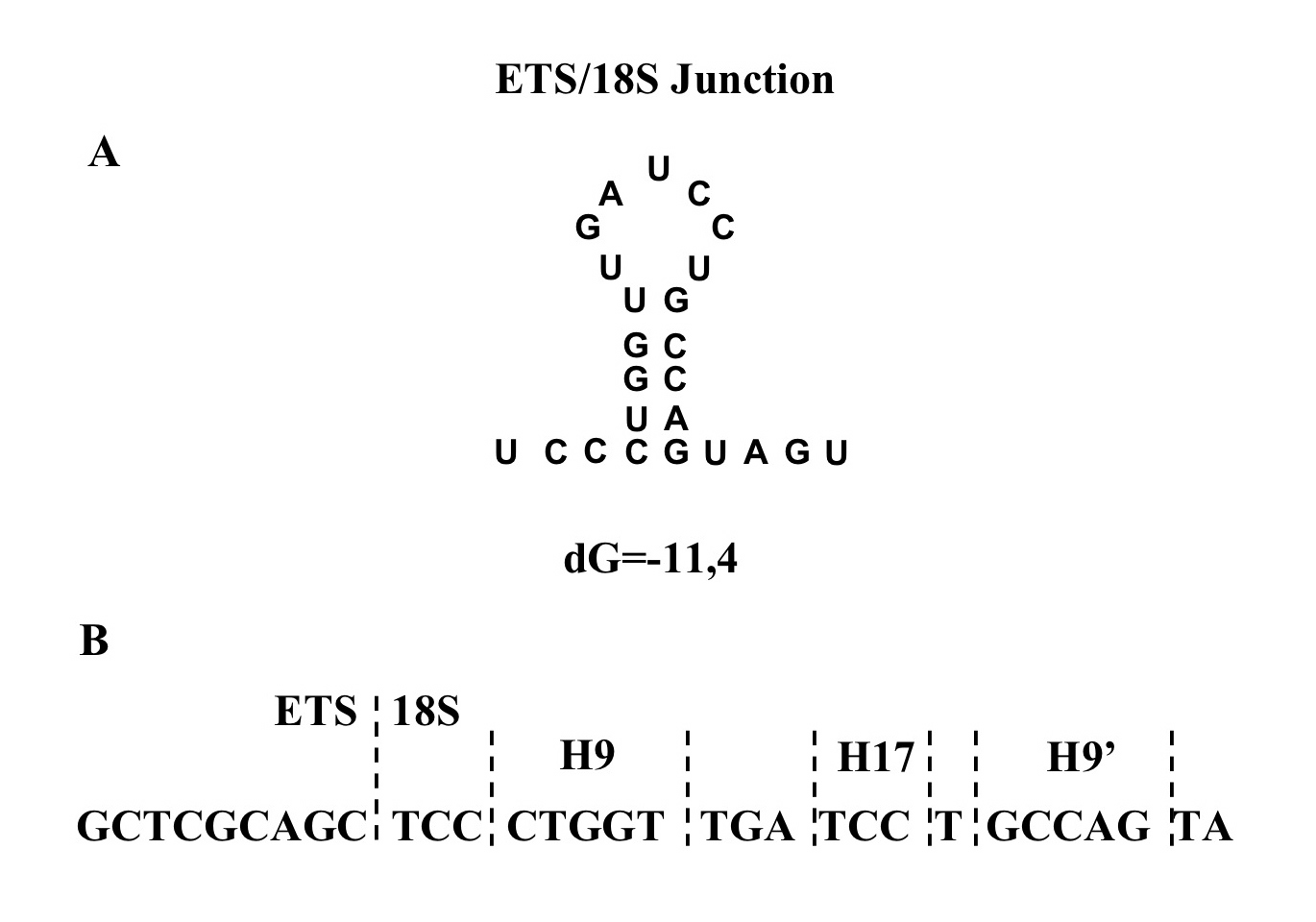

Supplement: Additional file 4 — Secondary structure of the ETS-18S junction. A) Secondary structure of the ETS-18S junction. Calculated free energy is reported below the structure. B) Conserved rRNA helices (H9, H17 and H9') flanking 3'-end ETS are within dashed vertical line [file 1471-2148-8-278-S4.jpeg]
